# Supplementary material for: Fungal small RNAs ride in extracellular vesicles to enter plant cells through clathrin-mediated endocytosis
Source: Nat Commun. 2023 Jul 20;14:4383. doi: 10.1038/s41467-023-40093-4 (PMC10359353; doi:10.1038/s41467-023-40093-4)

Supplementary Information

This PDF file includes:
Supplementary Figure 1 to 13
Supplementary Figure Legends

Supplementary Figure 1

a

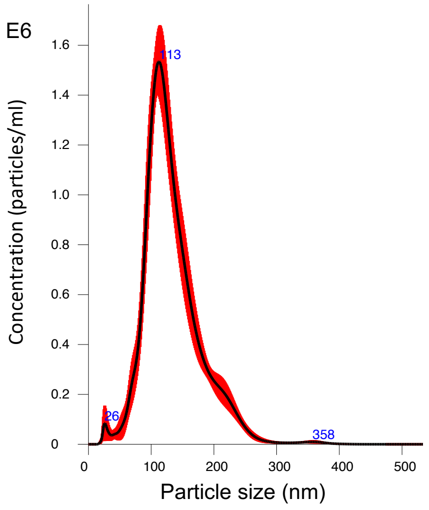

b

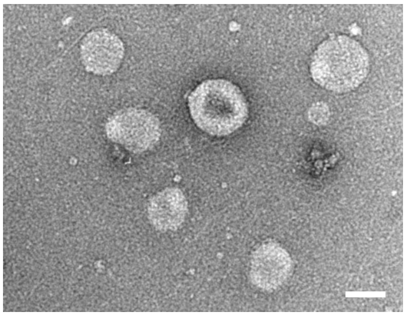

Supplementary Figure 1. Characterization of *B. cinerea* EVs.

a, The concentration and size distribution of EVs isolated from the supernatants of *B. cinerea* wild-type strain B05.10 liquid culture were determined using nanoparticle tracking analysis. The data of 4 measurements in one replicate was presented here. Similar data were obtained in three biological replicates. The red area represents the error bars. **b**, The isolated *B. cinerea* EVs were observed using transmission electron microscopy. Scale bar, 100nm.

Supplementary Figure 2

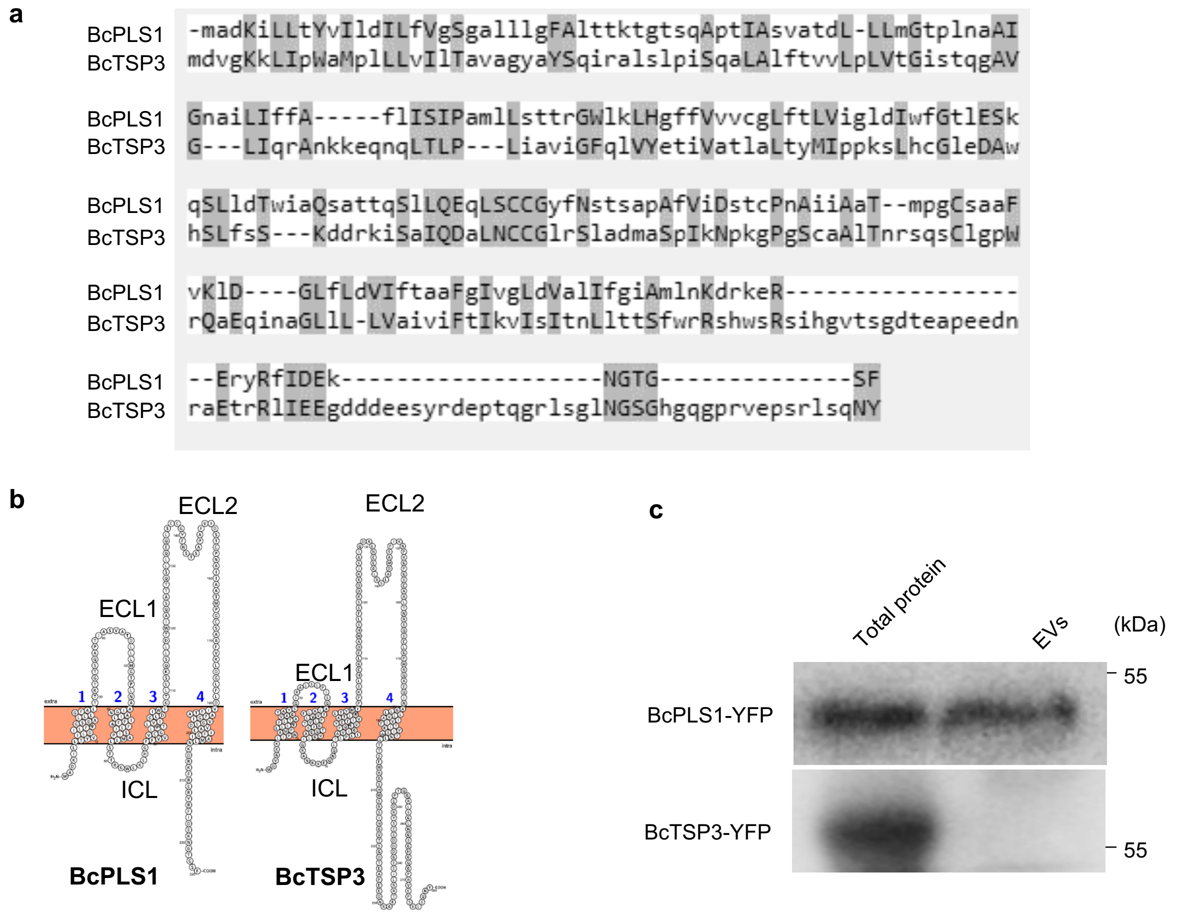

Supplementary Figure 2. Two tetraspanin proteins were found in *B. cinerea.*

a, Sequence alignment of BcPLS1 and BcTSP3. **b**, The predicted structures and the topology of BcPLS1 and BcTSP3. Images were made using Protter (https://wlab.ethz.ch/protter/start/). ECL1, small extracellular loop. ECL2, large extracellular loop. ICL, intracellular loop. **c**, BcPLS1-YFP protein can be detected in EV fractions isolated from liquid culture. Source data are provided as a Source Data file.

Supplementary Figure 3

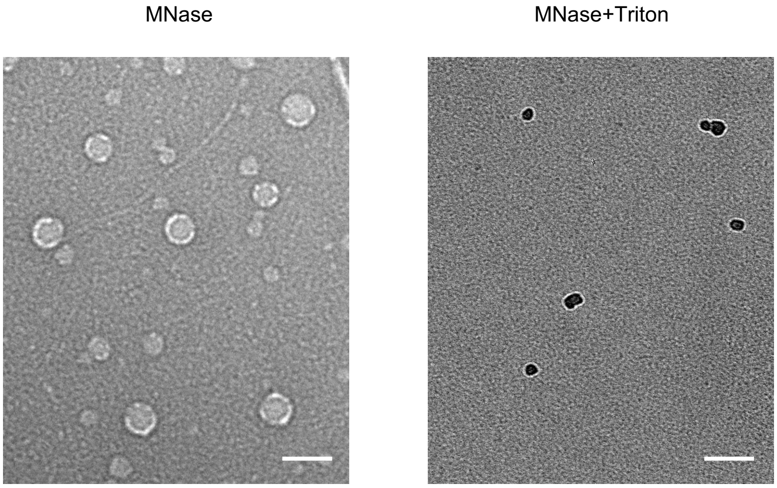

Supplementary Figure 3. ***B. cinerea*** **EVs are intact after MNase treatment.**

Transmission electron microscopy analysis demonstrated that *B. cinerea* extracellular vesicles (EVs) remained intact following MNase treatment. However, the addition of Triton X-100 to the reaction led to the rupture of *B. cinerea* EVs. Scale bar, 100nm.

Supplementary Figure 4

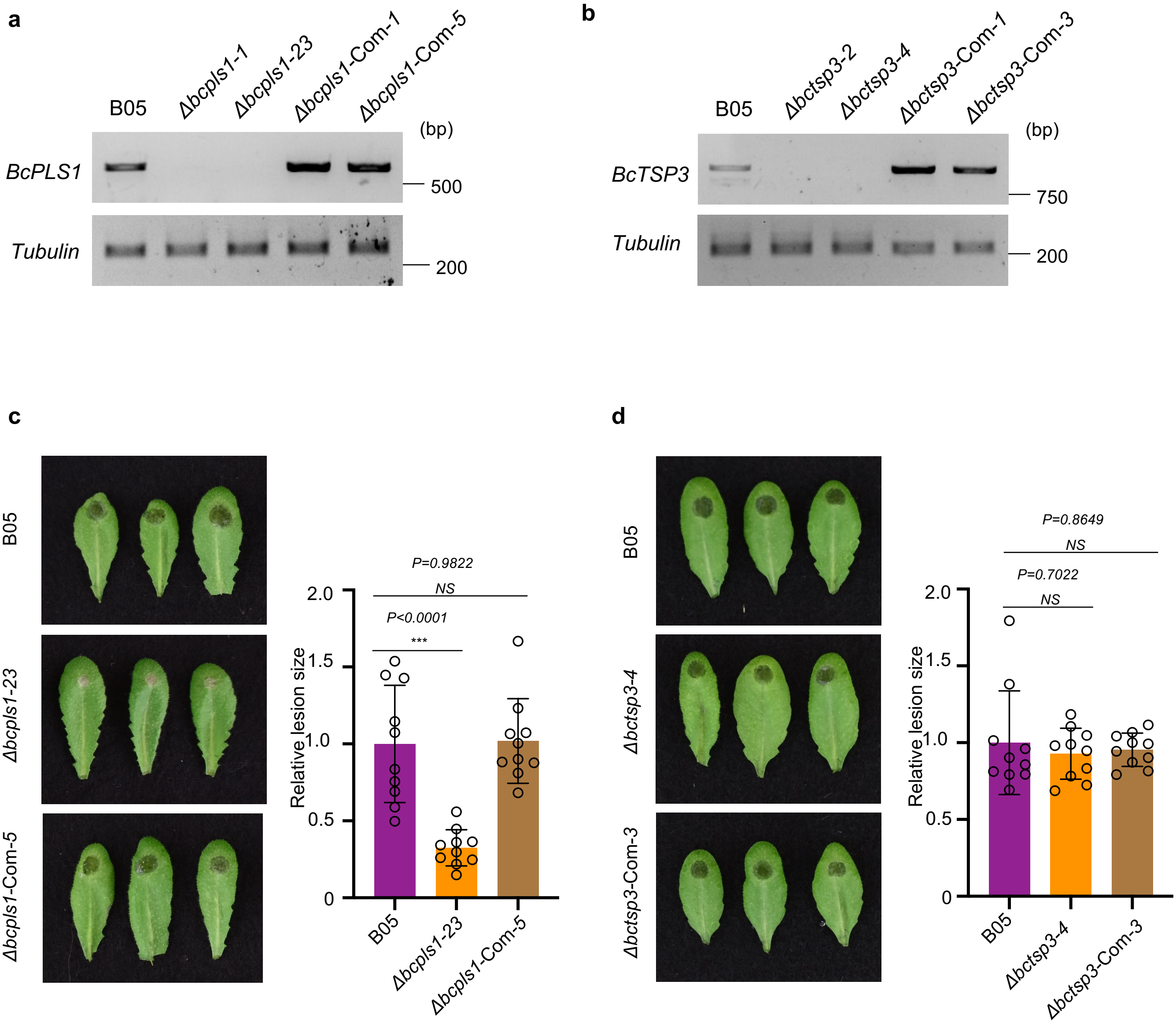

Supplementary Figure 4. Validation of *Δbcpls1* and *Δbctsp3* mutant and complementary strains

a, **b**, *BcPLS1* (**a**) and *BcTSP3* (**b**) expression was examined in B05, *Δbcpls1, Δbctsp3, Δbcpls1* complementary *and Δtsp3* complementary strains using RT-PCR. The *Tubulin* gene of *B. cinerea* was used as a control. **c**, **d**, Virulence of *Δbcpls1, Δbctsp3, Δbcpls1* complementary *and Δbctsp3* complementary strains was tested on wild-type *Arabidopsis* plants. Relative lesion size was determined by comparing it to the size of the entire leaf 2 days after infection. The statistical data are presented as mean ± s.d., *n* = 10 biological replicates. The statistical analysis was performed using ANOVA Dunnett's multiple comparisons test. The small open circles represent individual values. The error bars indicate s.d. ***$P < 0.001$. NS, not significant. Source data are provided as a Source Data file.

Supplementary Figure 5

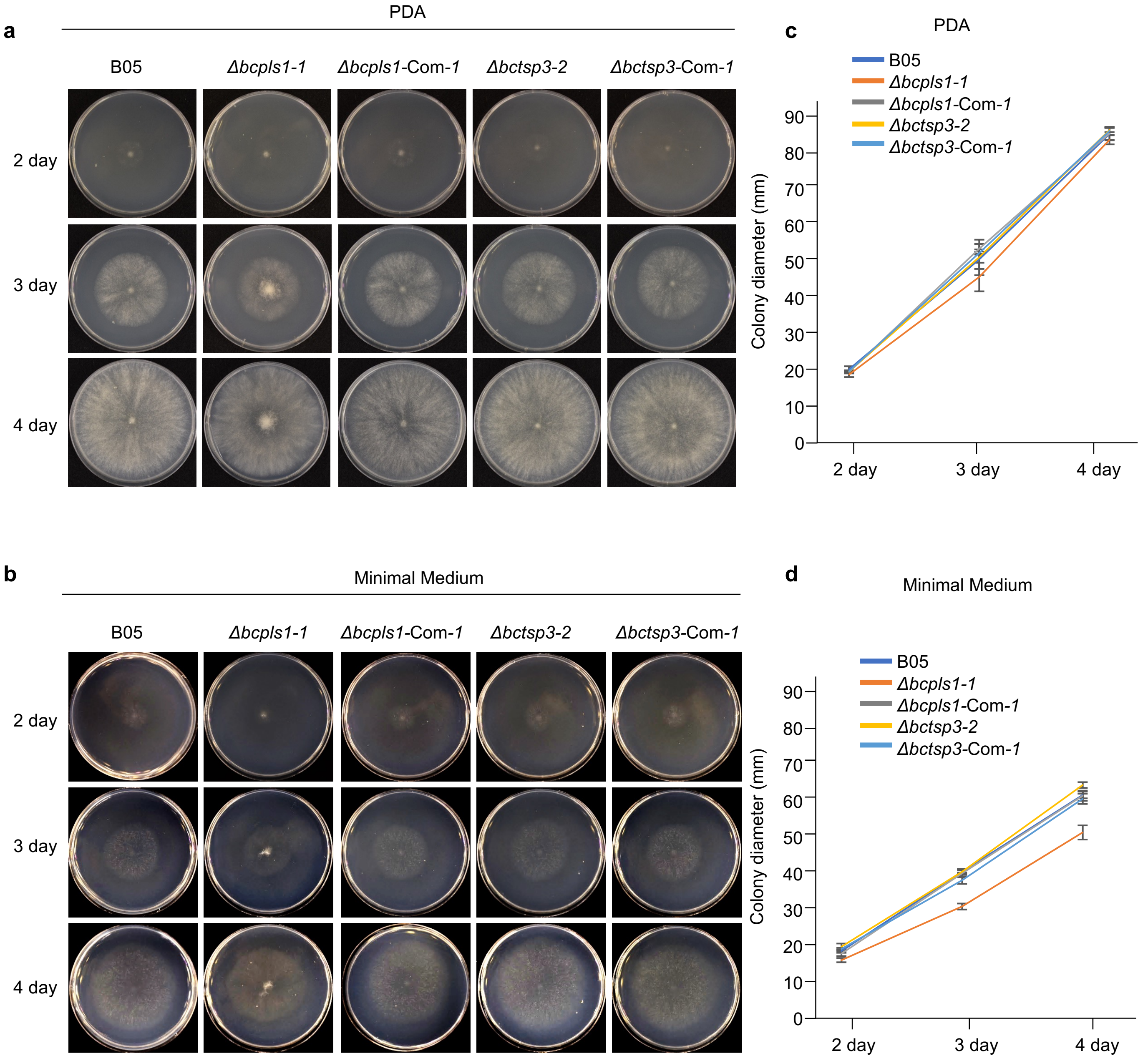

Supplementary Figure 5. Growth rate of B05, *Δbcpls1, Δbctsp3, Δbcpls1* complementary *and Δbctsp3* complementary strains on PDA and minimal medium plates

a, **b**, Growth rate of B05, *Δbcpls1, Δbctsp3, Δbcpls1* complementary *and Δbctsp3* complementary strains on PDA (**a**) and minimal medium (**b**) from day 2 to day 4. 10 μl of 10^5 spores were cultured to measure the growth rate of each strain at room temperature. **c**, **d**, The quantification of colony diameters of each strain on PDA (**c**) and minimal medium (**d**). The data were presented as mean ± s.d., *n* = 4 biological replicates. Source data are provided as a Source Data file.

Supplementary Figure 6

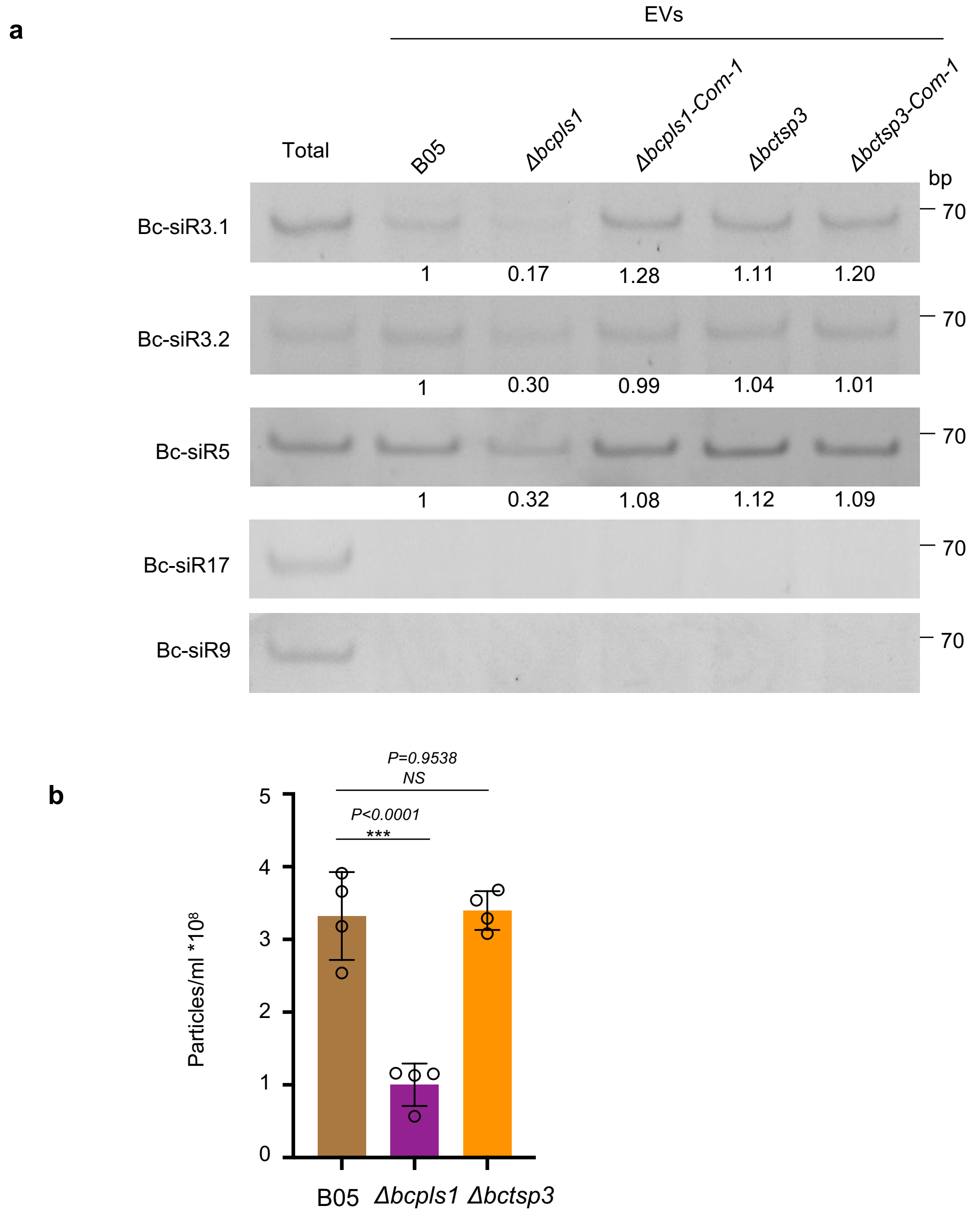

Supplementary Figure 6. Bc-sRNA amount is reduced in EVs from the *Δbcpls1* mutant strain.

a, Bc-sRNAs were examined in EVs isolated from *Δbcpls1, Δbctsp3, Δbcpls1* complementary *and Δbctsp3* complementary strains using sRNA RT-PCR. Bc-siR17 and Bc-siR9 were used as negative control. **b**, EV amount from *Δbcpls1* was reduced compared to wild-type strain B05 and *Δbctsp3* mutant. The statistical data are presented as mean ± s.d., $n = 4$ measurements. The statistical analysis was performed using ANOVA Dunnett's multiple comparisons test. The small open circles represent individual values. The error bars indicate s.d. $^{**}P < 0.01$, $^{***}P < 0.001$. NS, not significant. Source data are provided as a Source Data file.

Supplementary Figure 7

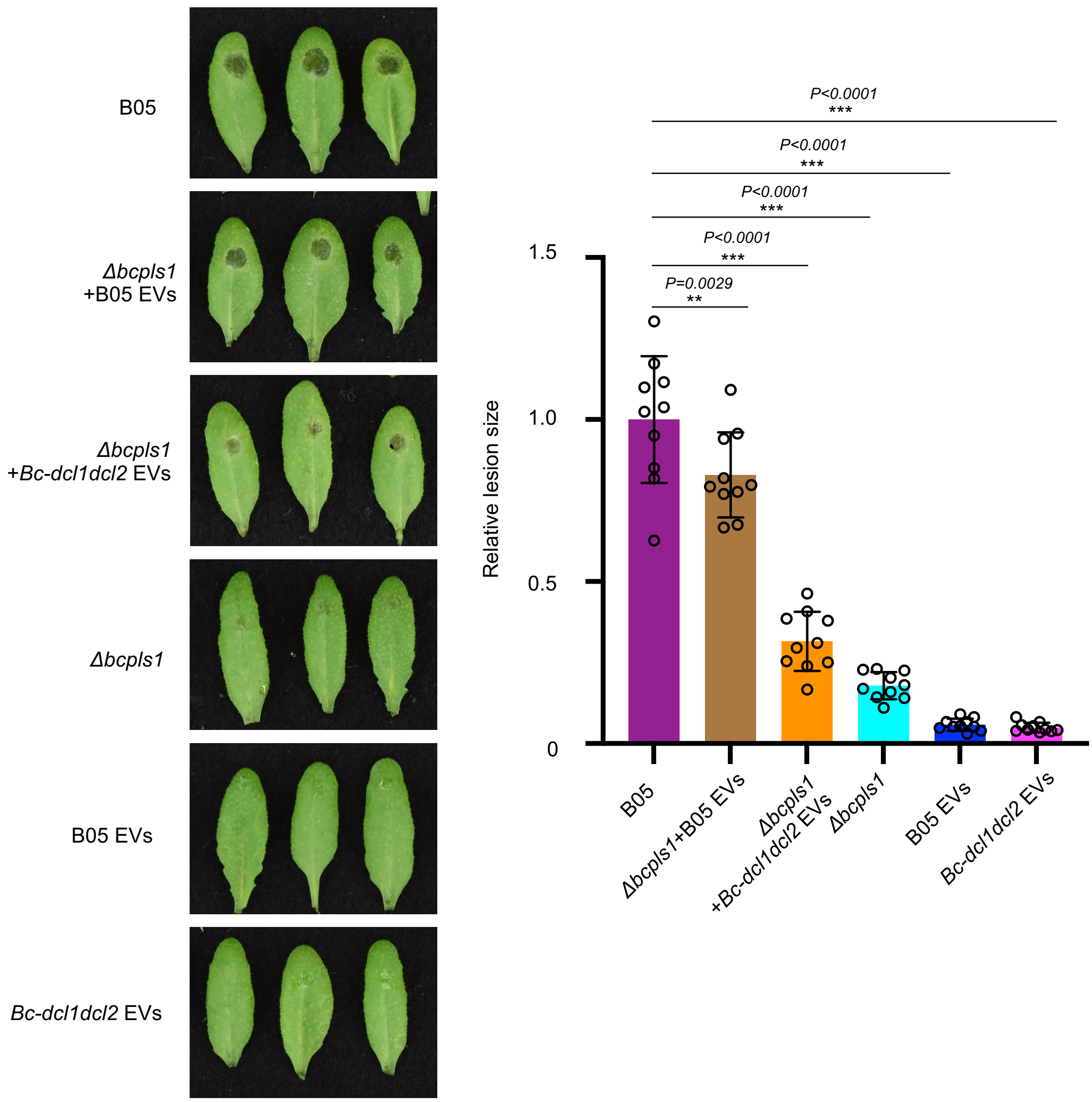

Supplementary Figure 7. EVs from the *bcdcl1dcl2* mutant weakly complement the virulence of the *Δbcpls1* mutant strain*.*

EVs from *B. cinerea* wild-type or *bcdcl1dcl2* mutant strains were premixed with *Δbcpls1* spores to detect the virulence of *Δbcpls1* on *Arabidopsis* plants*.* Relative lesion size was determined by comparing it to the size of the entire leaf 2 days after infection. The statistical data are presented as mean ± s.d., $n = 10$ biological replicates. The statistical analysis was performed using ANOVA Dunnett's multiple comparisons test. The small open circles represent individual values. The error bars indicate s.d. ***$P < 0.001$. Source data are provided as a Source Data file.

Supplementary Figure 8

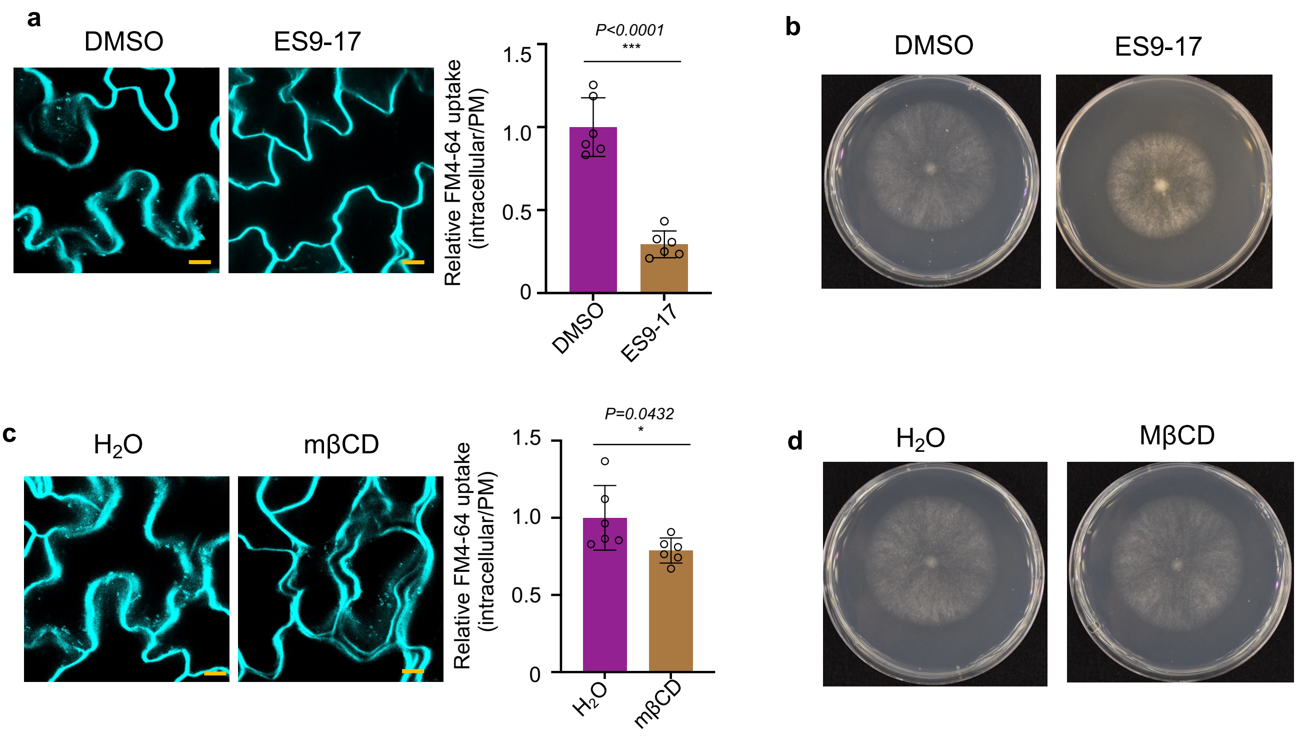

Supplementary Figure 8. Effect of ES9-17 and MβCD treatment on *Arabidopsis* plants and *B. cinerea*.

a, FM4-64 uptake of *Arabidopsis* leaves was examined after 30 μM ES9-17 treatment. **b,** The growth phenotype of wild-type *B. cinerea* B05 on PDA plate with 30 μM ES9-17 **c**, FM4-64 uptake of *Arabidopsis* leaves was examined after 10 mM mβCD treatment. **d**, The growth phenotype of wild-type *B. cinerea* B05 on PDA plate with 10 mM mβCD. The uptake efficacy in (**a**) and (**c**) was measured by calculating the signal ratio between intracellular and plasma membrane (PM) intensities. The statistical data are presented as mean ± s.d., $n = 6$ independent pictures in (**a**) and (**c**). The statistical analysis was performed using unpaired two-tailed Student's t-test. The small open circles represent individual values. The error bars indicate s.d. $^*P < 0.05$, $^{**}P < 0.01$, $^{***}P < 0.001$. Source data are provided as a Source Data file.

Supplementary Figure 9

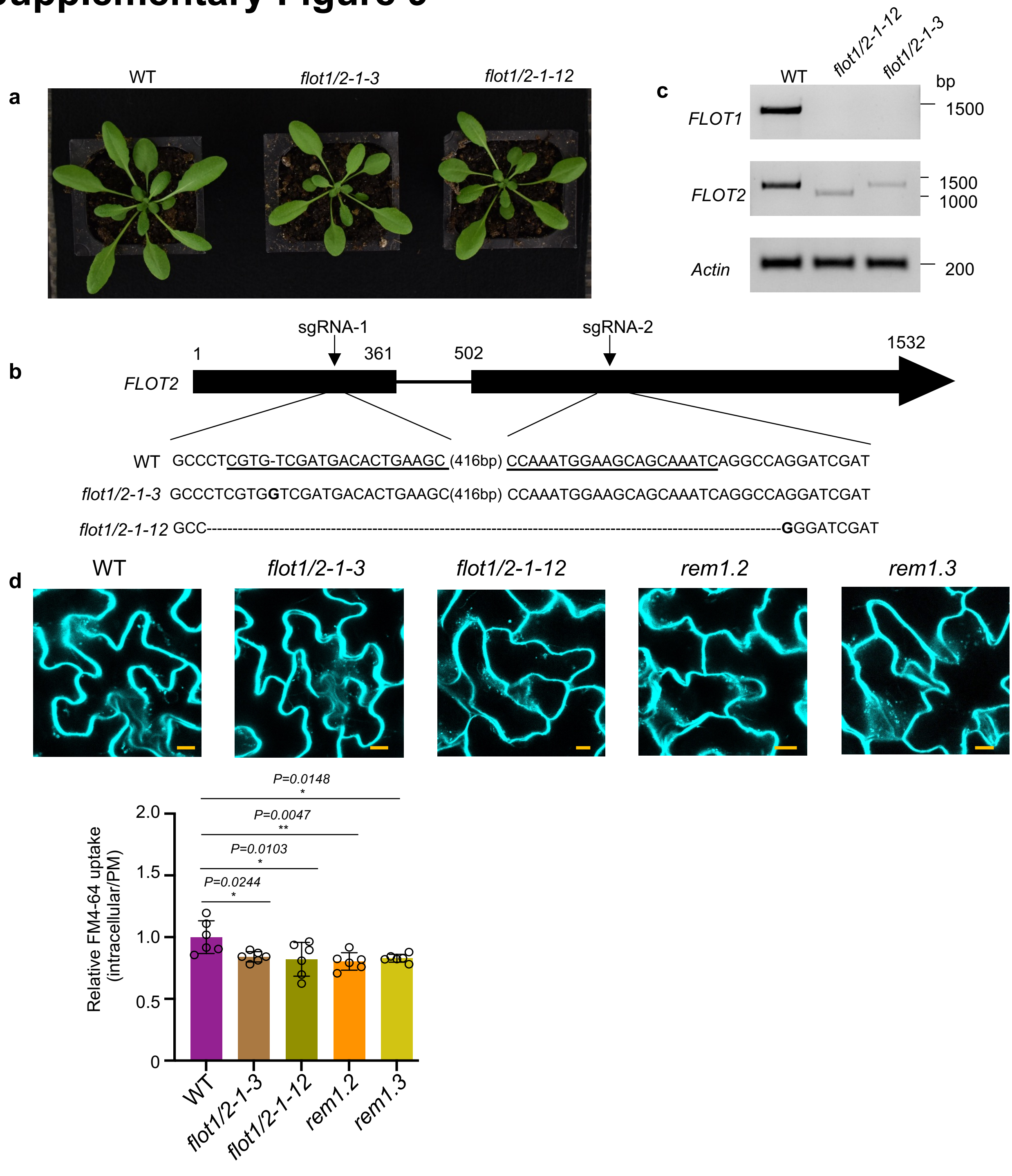

Supplementary Figure 9. Validation of *Arabidopsis flot1/2* mutant lines.
a, Phenotypes of wild-type and two *flot1/2* homozygous mutants of 28-d-old plants. **b**, CRISPR Cas9-mutagenized DNA sequences of *FLOT2* in two homozygous mutants. *flot1/2-1-3* is a frameshift mutant with single nucleotide insertion. *flot1/2-1-12* is a deletion mutant with 464 bp removed from the *FLOT2* gene. sgRNA sequences were underlined in the WT *FLOT2* sequence. **c**, Expression of *FLOT1* and *FLOT2* in mutant lines were examined using RT-PCR. Actin was used as a control. **d,** FM4-64 uptake was examined in *flot1/2* double mutants, *rem1.2*, *rem1.3* mutants. The uptake efficacy was measured by calculating the signal ratio between intracellular and plasma membrane (PM) intensities. The statistical data are presented as mean ± s.d., $n = 6$ independent pictures. The statistical analysis was performed using ANOVA Dunnett's multiple comparisons test. The small open circles represent individual values. The error bars indicate s.d. $*P < 0.05$, $**P < 0.01$, $***P < 0.001$. Source data are provided as a Source Data file.

Supplementary Figure 10

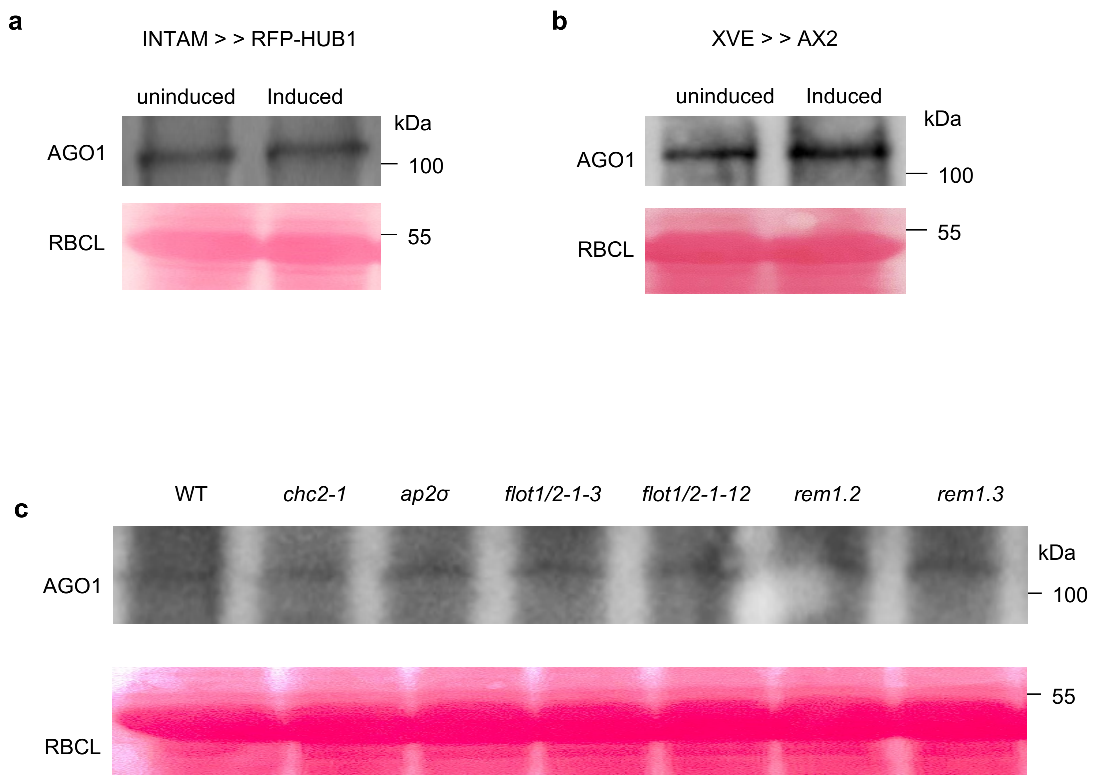

Supplementary Figure 10. The amount of Arabidopsis AGO1 protein is not affected by different mutants.

a,b, The total amount of AGO1 protein in uninduced and induced INTAM > > RFP-HUB1 (**a**) and XVE > > AX2 (**b**) lines were examined using western blot. Ponceau-S staining of Rubisco was used as the loading control. **c**, The total amount of AGO1 protein in WT, *chc2-1*, *ap2σ, flot1/2-1-3, flot1/2-1-12, rem1.2 and rem1.3* were examined using western blot. Ponceau-S staining of Rubisco was used as the loading control. Source data are provided as a Source Data file.

Supplementary Figure 11

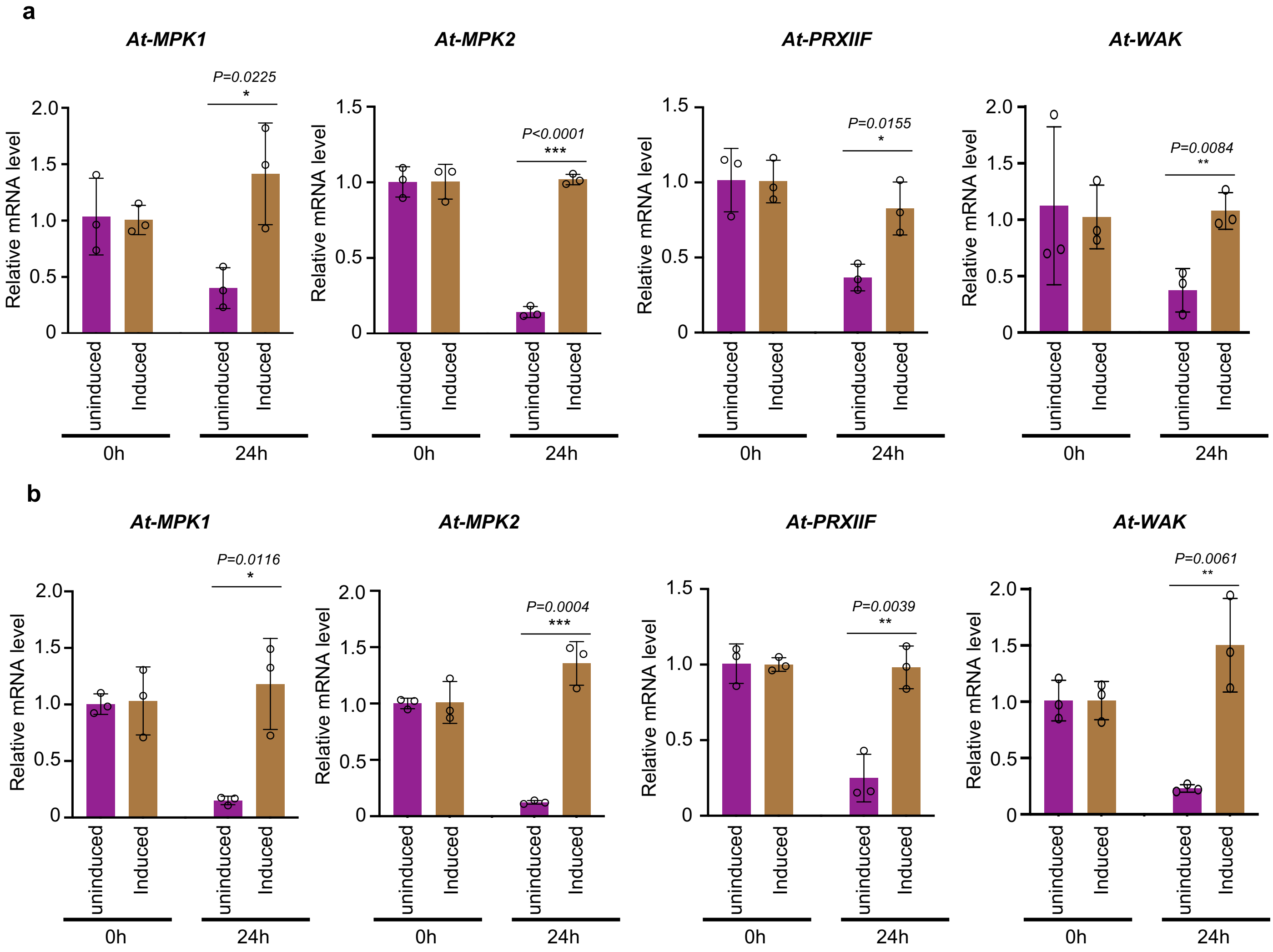

Supplementary Figure 11. Suppression of host target genes by Bc-sRNAs was attenuated in induced INTAM > > RFP-HUB1 and XVE > > AX2 lines.

a, b, The host target genes suppression of Bc-sRNAs was attenuated in induced INTAM > > RFP-HUB1 (**a**) and XVE > > AX2 (**b**) lines. The expression of Bc-siR3.1 target *At-PRXIIF*, Bc-siR3.2 target *At-MPK1* and *At-MPK2*, and Bc-siR5 target *At-WAK* in induced INTAM > > RFP-HUB1 and XVE > > AX2 lines compared with those uninduced plants after *B. cinerea* infection was examined by real-time RT-PCR. The data are presented as mean ± s.d. Similar results were obtained in three biologically independent experiments. The statistical analysis was performed using unpaired two-tailed Student's *t*-test. The small open circles represent the individual values. The error bars indicate s.d. *$P < 0.05$, **$P < 0.01$, ***$P < 0.001$. Source data are provided as a Source Data file.

Supplementary Figure 12

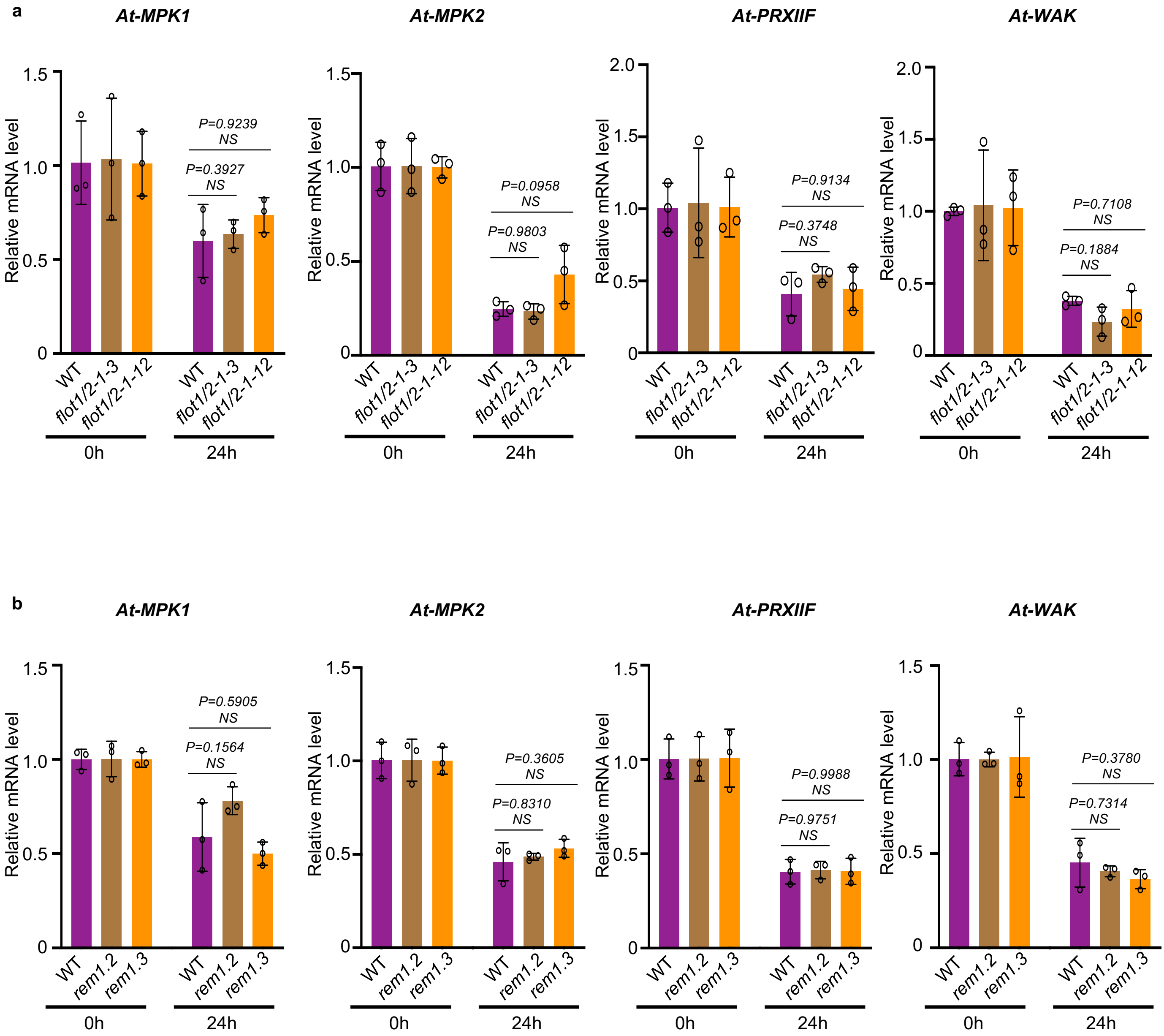

Supplementary Figure 12. Suppression of host target genes by Bc-sRNAs was not affected in clathrin-independent endocytosis mutants

a,b, The expression of Bc-siR3.1 target *At-PRXIIF*, Bc-siR3.2 target *At-MPK1* and *At-MPK2*, and Bc-siR5 target *At-WAK* in *flot1/2* double mutants **(a)** and *rem1.2, rem1.3* **(b)** mutants compared with those in wild-type plants after *B. cinerea* infection was examined by real-time RT-PCR. The data are presented as mean ± s.d. Similar results were obtained in three biologically independent experiments. The statistical analysis was performed using ANOVA Dunnett's multiple comparisons test. The small open circles represent the individual values. The error bars indicate s.d. **P* < 0.05, ***P* < 0.01, ****P* < 0.001. NS, not significant. Source data are provided as a Source Data file.

Supplementary Figure 13

Results for biological replicates conducted in this study with corresponding figure numbers indicated. Source data are provided as a Source Data file.

Fig. 1c

Replicate 2

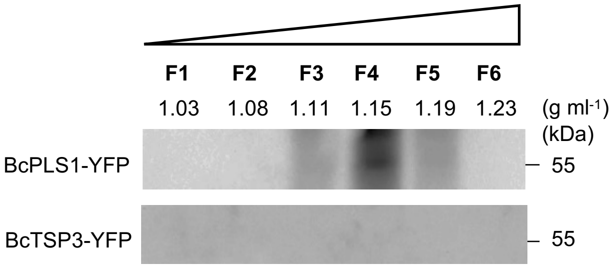

Replicate 3

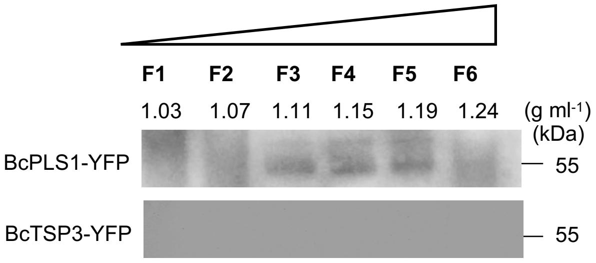

Fig. 1f

Replicate 2

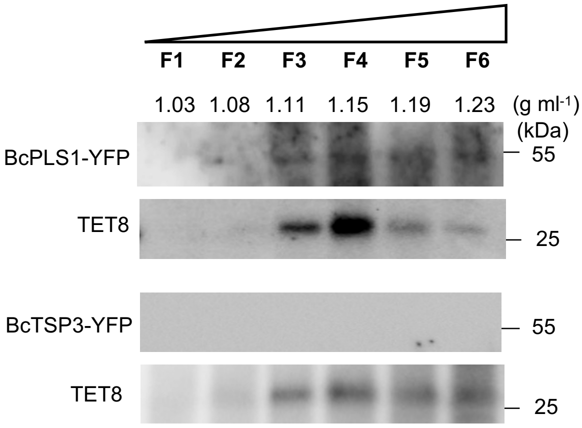

Replicate 3

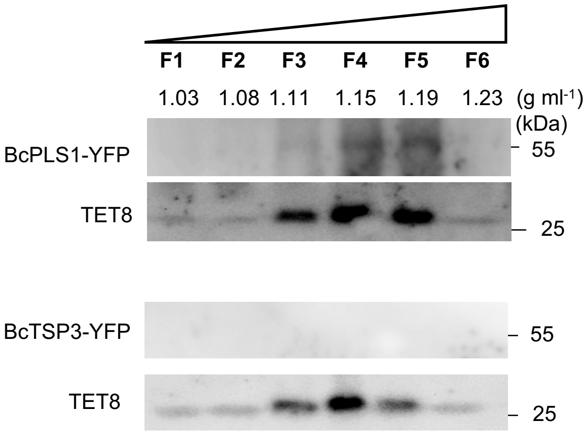

Fig. 2a

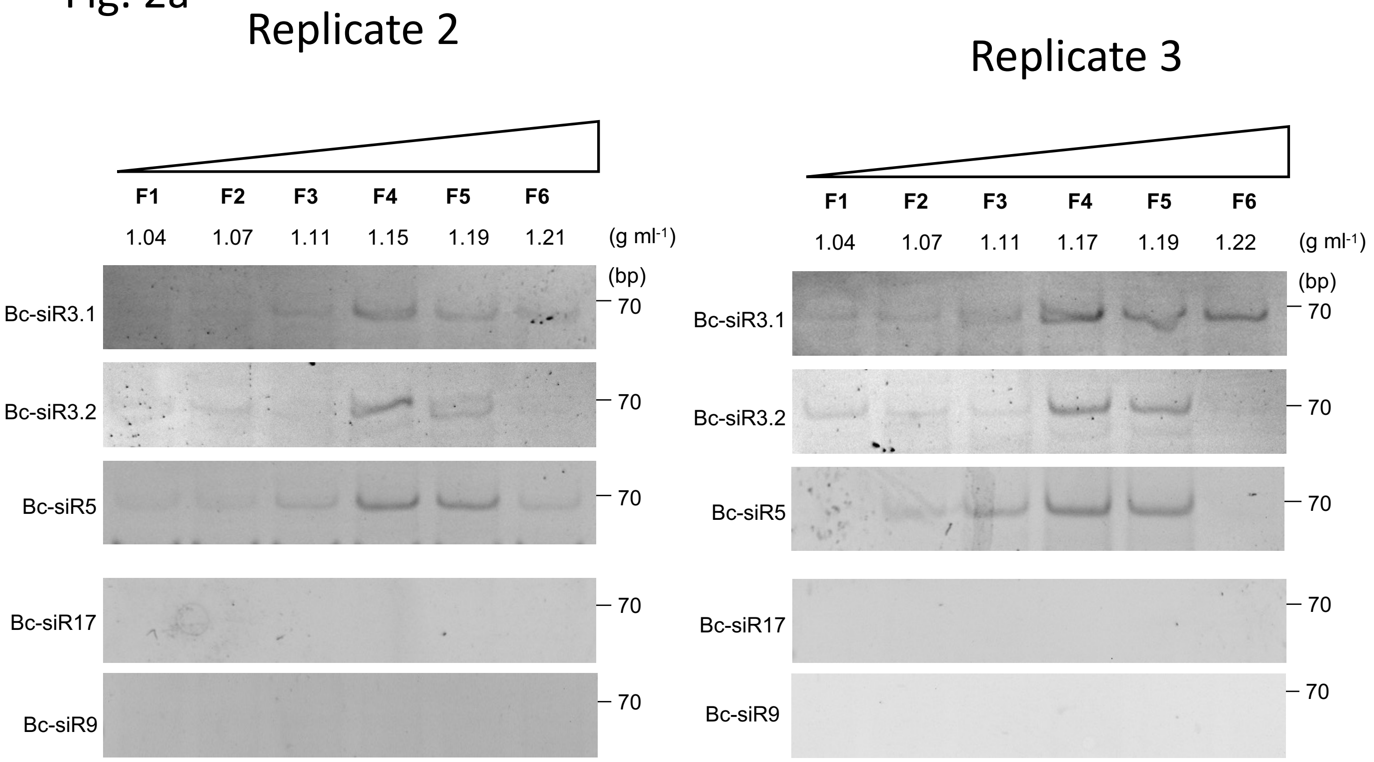

Replicate 2
F1
F2
F3
F4
F5
F6
1.04
1.07
1.11
1.15
1.19
1.21
(g ml-1)
(bp)
Bc-siR3.1
Bc-siR3.2
Bc-siR5
Bc-siR17
Bc-siR9
70
Replicate 3
F1
F2
F3
F4
F5
F6
1.04
1.07
1.11
1.17
1.19
1.22
(g ml-1)
(bp)
Bc-siR3.1
Bc-siR3.2
Bc-siR5
Bc-siR17
Bc-siR9
70

Fig. 2b

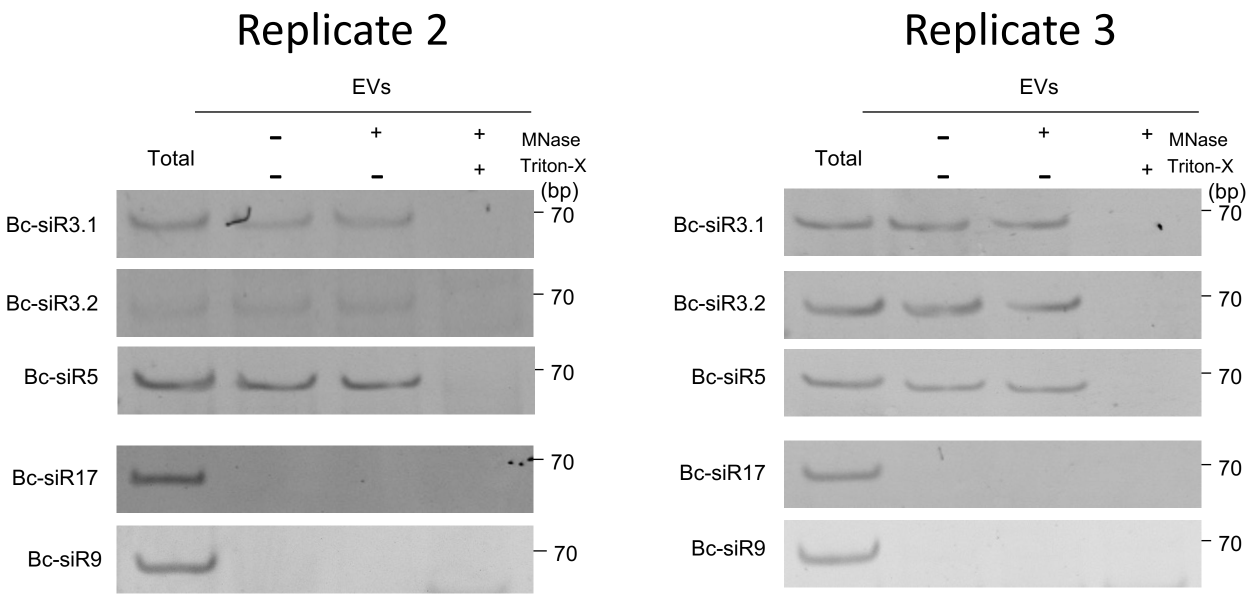

Replicate 2
EVs
Total
MNase
Triton-X
(bp)
Bc-siR3.1
Bc-siR3.2
Bc-siR5
Bc-siR17
Bc-siR9
70
Replicate 3
EVs
Total
MNase
Triton-X
(bp)
Bc-siR3.1
Bc-siR3.2
Bc-siR5
Bc-siR17
Bc-siR9
70

Fig. 4a

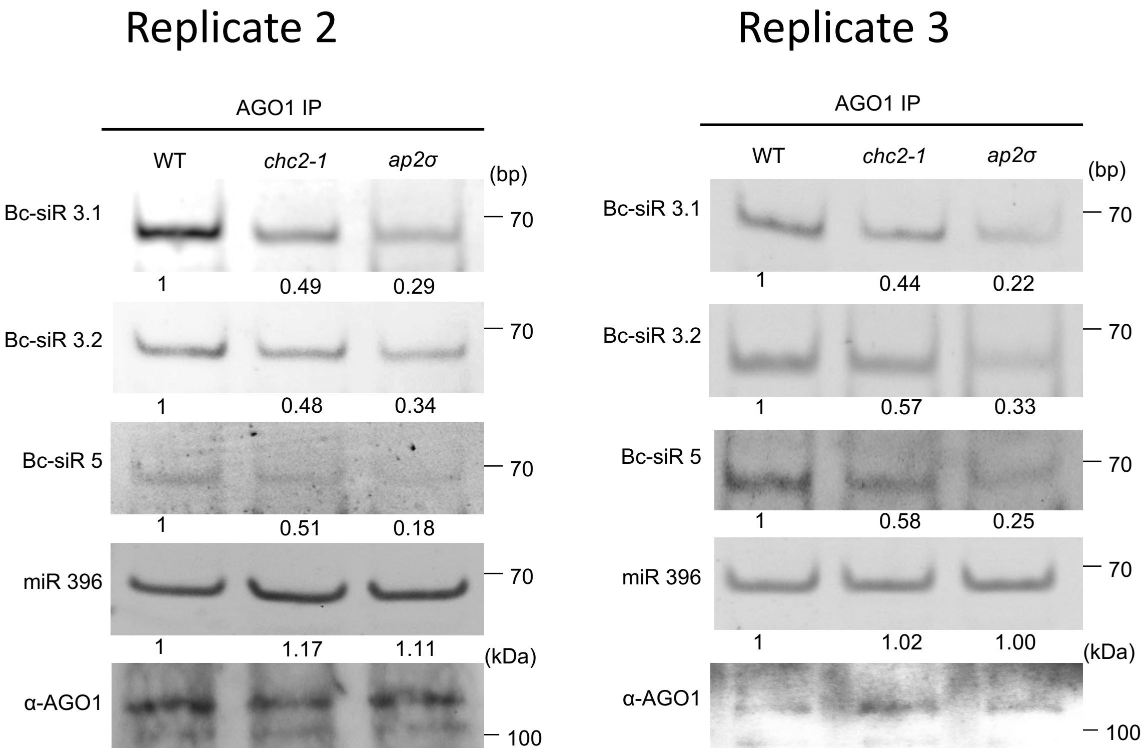
Replicate 2
AGO1 IP
WT
chc2-1
ap2σ
(bp)
Bc-siR 3.1
70
1
0.49
0.29
Bc-siR 3.2
70
1
0.48
0.34
Bc-siR 5
70
1
0.51
0.18
miR 396
70
1
1.17
1.11
(kDa)
α-AGO1
100
Replicate 3
AGO1 IP
WT
chc2-1
ap2σ
(bp)
Bc-siR 3.1
70
1
0.44
0.22
Bc-siR 3.2
70
1
0.57
0.33
Bc-siR 5
70
1
0.58
0.25
miR 396
70
1
1.02
1.00
(kDa)
α-AGO1
100

Fig. 4b

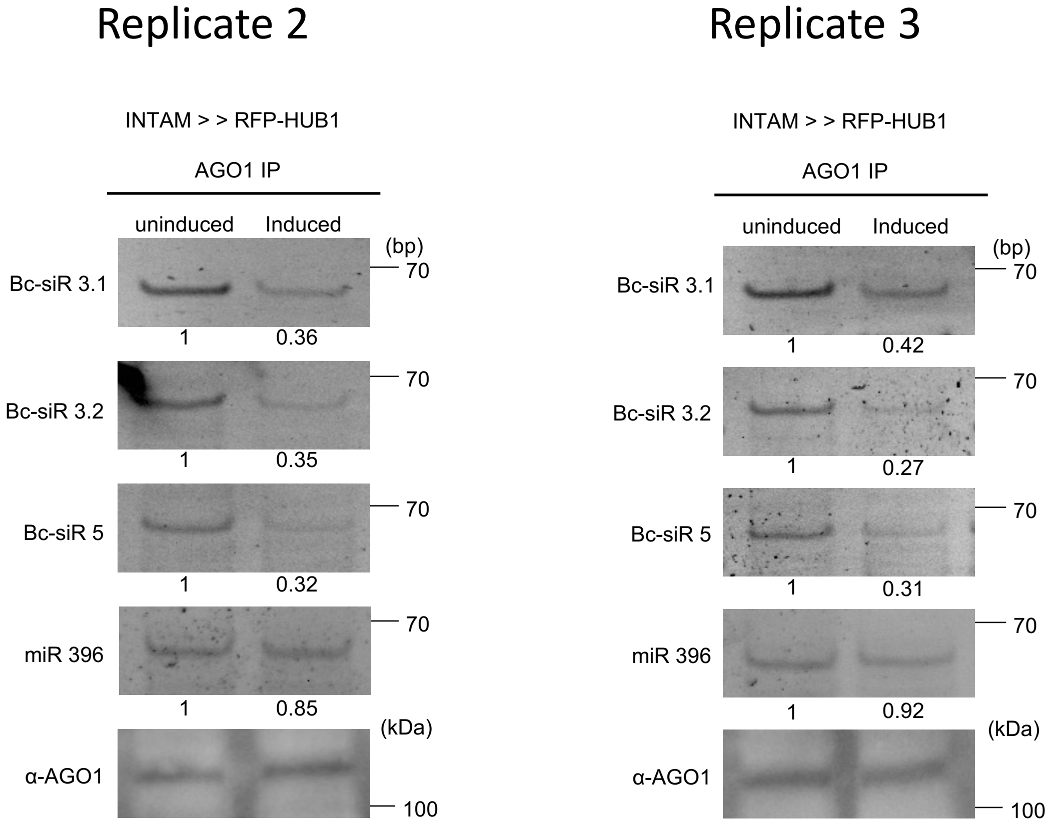
Replicate 2
INTAM > > RFP-HUB1
AGO1 IP
uninduced
Induced
(bp)
Bc-siR 3.1
70
1
0.36
Bc-siR 3.2
70
1
0.35
Bc-siR 5
70
1
0.32
miR 396
70
1
0.85
(kDa)
α-AGO1
100
Replicate 3
INTAM > > RFP-HUB1
AGO1 IP
uninduced
Induced
(bp)
Bc-siR 3.1
70
1
0.42
Bc-siR 3.2
70
1
0.27
Bc-siR 5
70
1
0.31
miR 396
70
1
0.92
(kDa)
α-AGO1
100

Fig. 4c

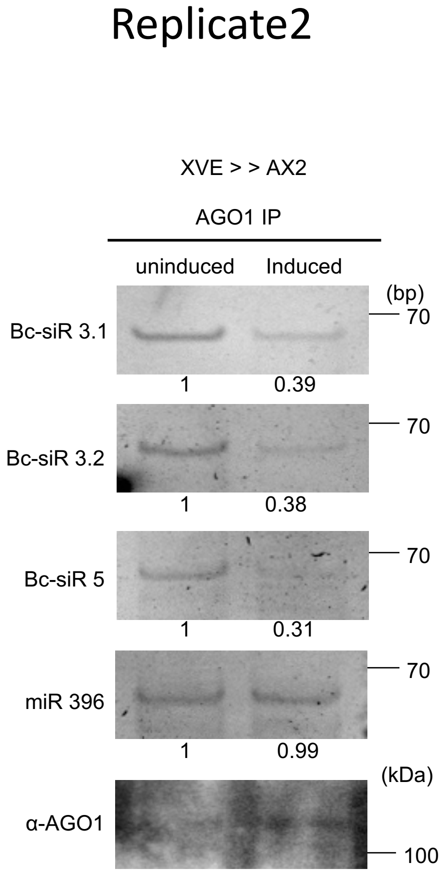
Replicate2
XVE > > AX2
AGO1 IP
uninduced
Induced
(bp)
Bc-siR 3.1
70
1
0.39
Bc-siR 3.2
70
1
0.38
Bc-siR 5
70
1
0.31
miR 396
70
1
0.99
(kDa)
α-AGO1
100

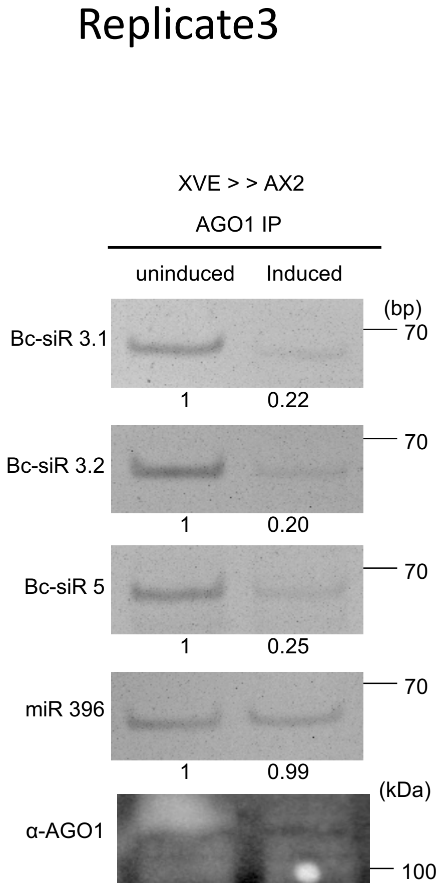
Replicate3
XVE > > AX2
AGO1 IP
uninduced
Induced
(bp)
Bc-siR 3.1
70
1
0.22
Bc-siR 3.2
70
1
0.20
Bc-siR 5
70
1
0.25
miR 396
70
1
0.99
(kDa)
α-AGO1
100

Fig. 4d

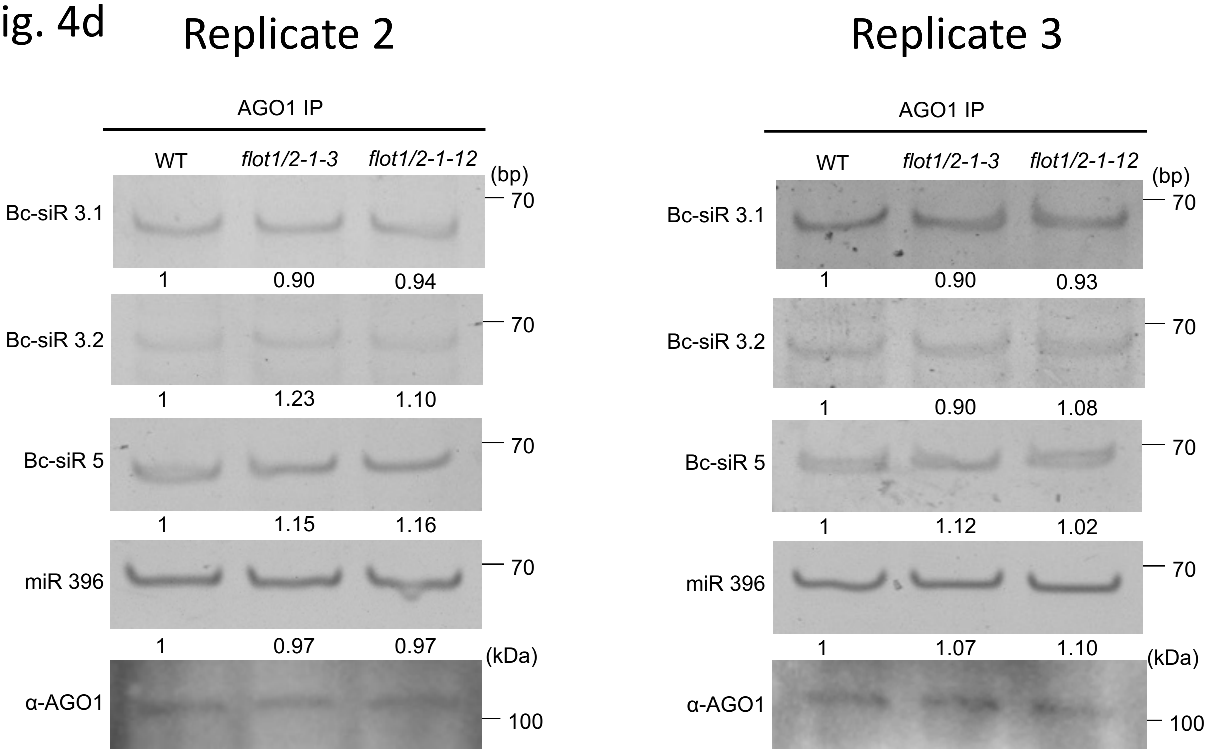
Replicate 2
AGO1 IP
WT
flot1/2-1-3
flot1/2-1-12
(bp)
Bc-siR 3.1
70
1 0.90 0.94
Bc-siR 3.2
70
1 1.23 1.10
Bc-siR 5
70
1 1.15 1.16
miR 396
70
1 0.97 0.97
(kDa)
α-AGO1
100
Replicate 3
AGO1 IP
WT
flot1/2-1-3
flot1/2-1-12
(bp)
Bc-siR 3.1
70
1 0.90 0.93
Bc-siR 3.2
70
1 0.90 1.08
Bc-siR 5
70
1 1.12 1.02
miR 396
70
1 1.07 1.10
(kDa)
α-AGO1
100

Fig. 4e

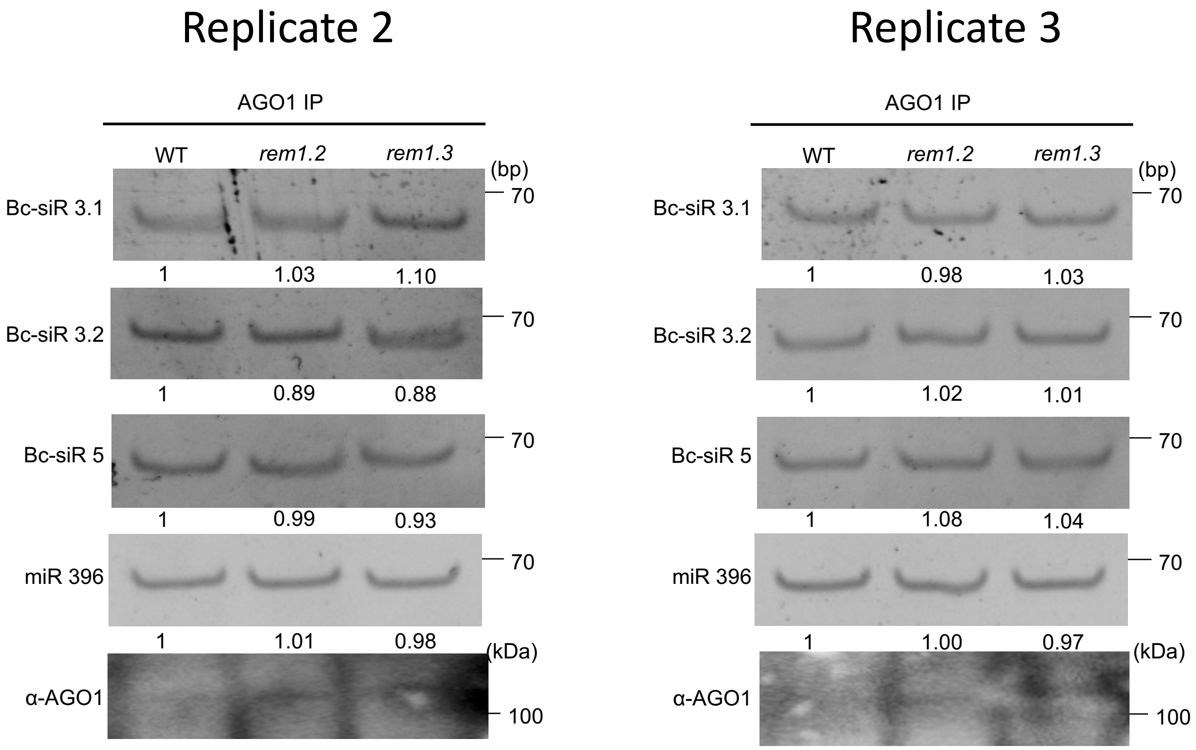
Replicate 2
AGO1 IP
WT
rem1.2
rem1.3
(bp)
Bc-siR 3.1
70
1 1.03 1.10
Bc-siR 3.2
70
1 0.89 0.88
Bc-siR 5
70
1 0.99 0.93
miR 396
70
1 1.01 0.98
(kDa)
α-AGO1
100
Replicate 3
AGO1 IP
WT
rem1.2
rem1.3
(bp)
Bc-siR 3.1
70
1 0.98 1.03
Bc-siR 3.2
70
1 1.02 1.01
Bc-siR 5
70
1 1.08 1.04
miR 396
70
1 1.00 0.97
(kDa)
α-AGO1
100

Fig. 5a

Replicate 2

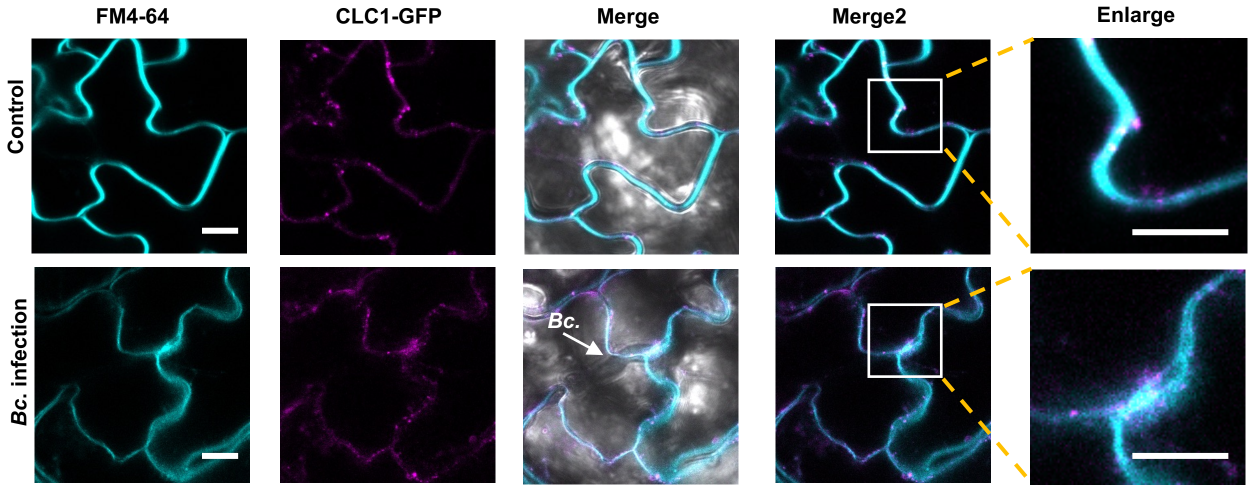

Replicate 3

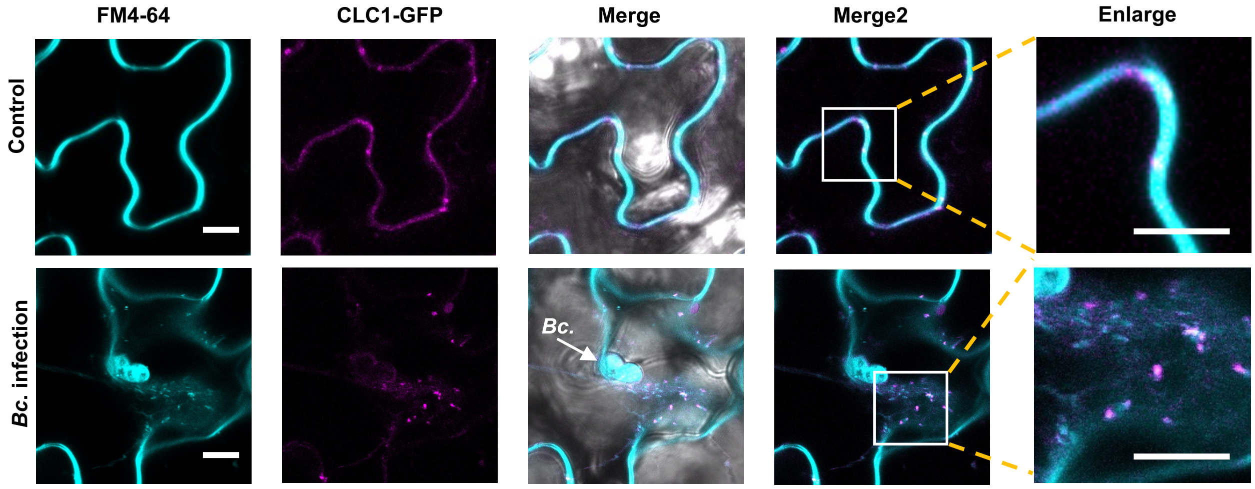

Fig. 5b

Replicate 2

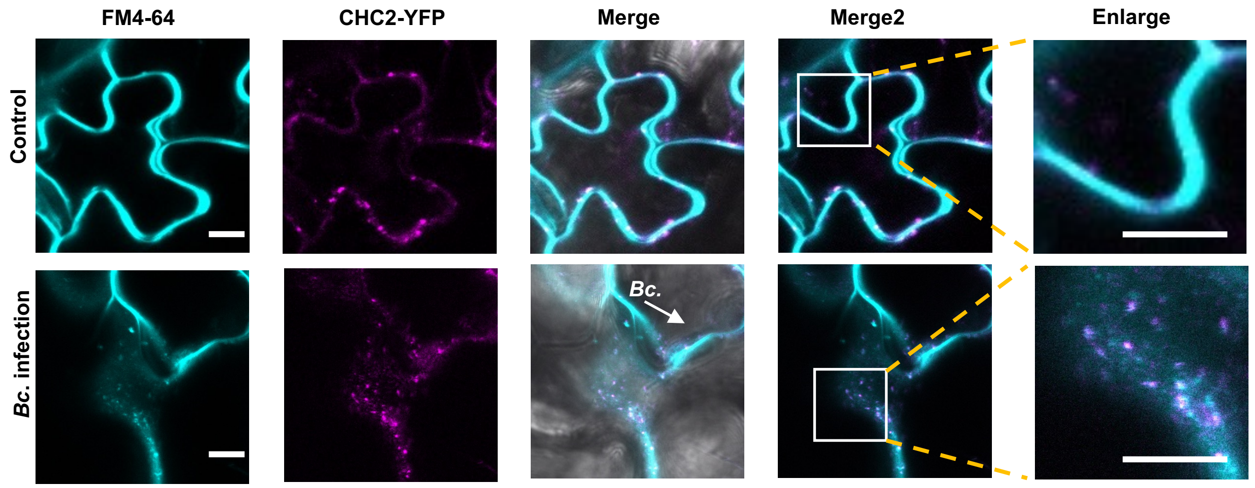

Replicate 3

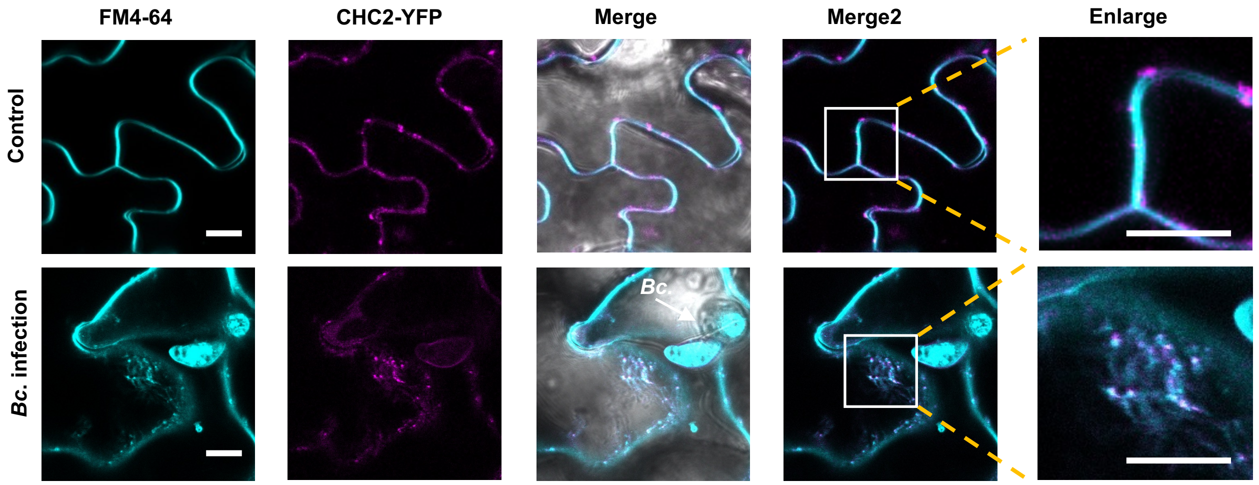

Fig. 5c

Replicate 2

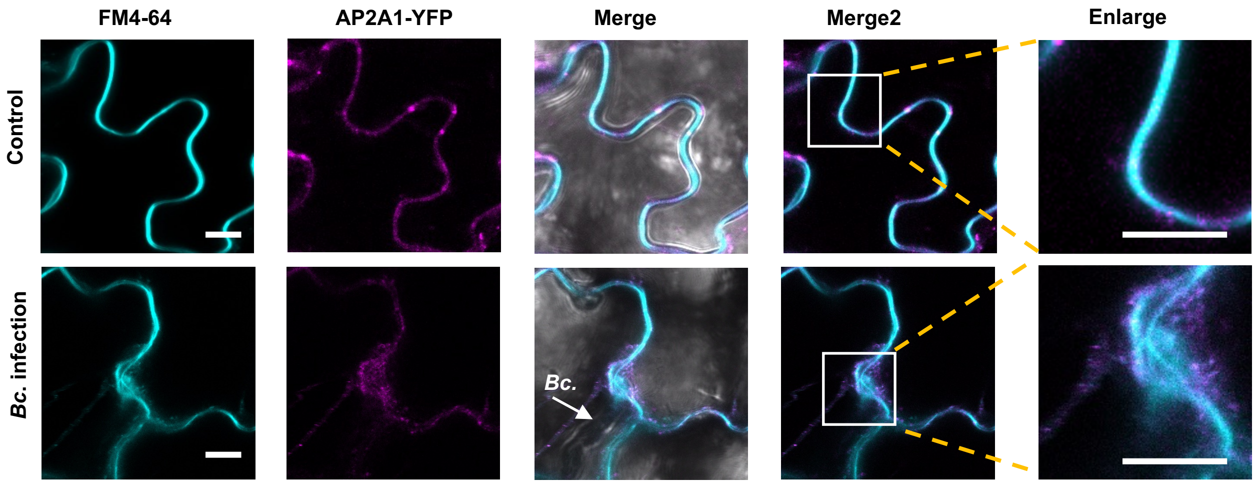

Replicate 3

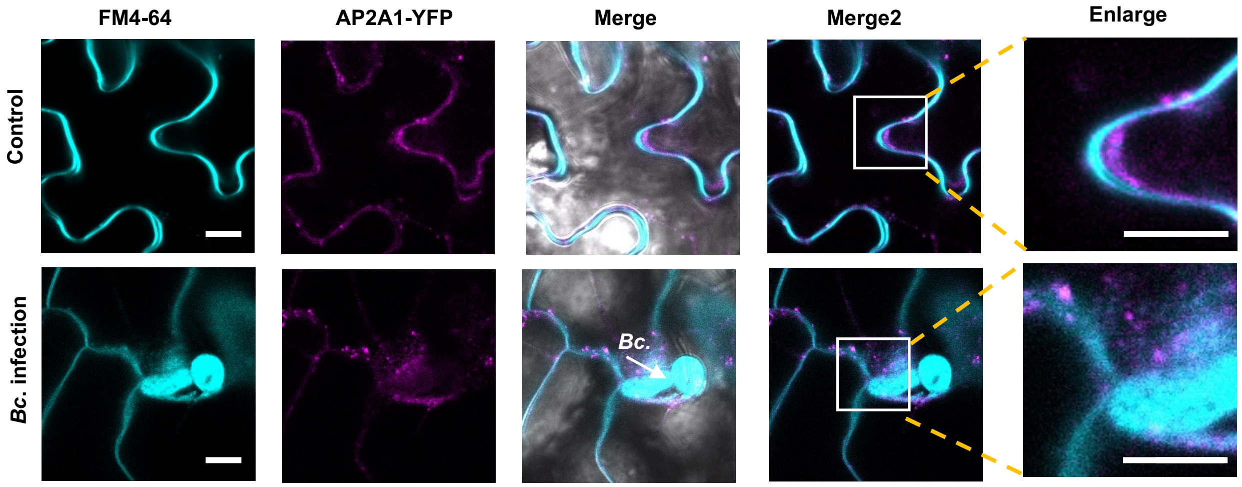

Fig. 5d

Replicate 2

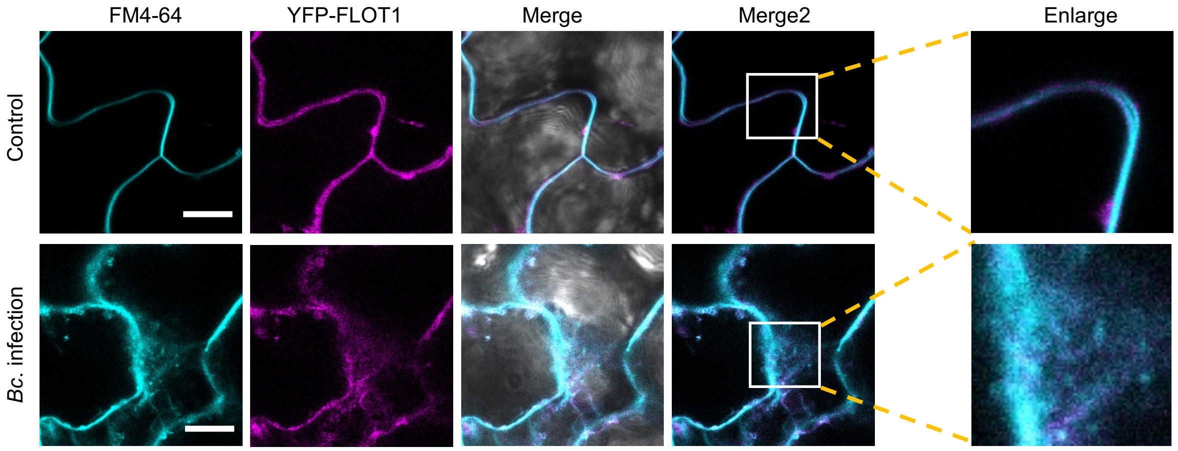

Replicate 3

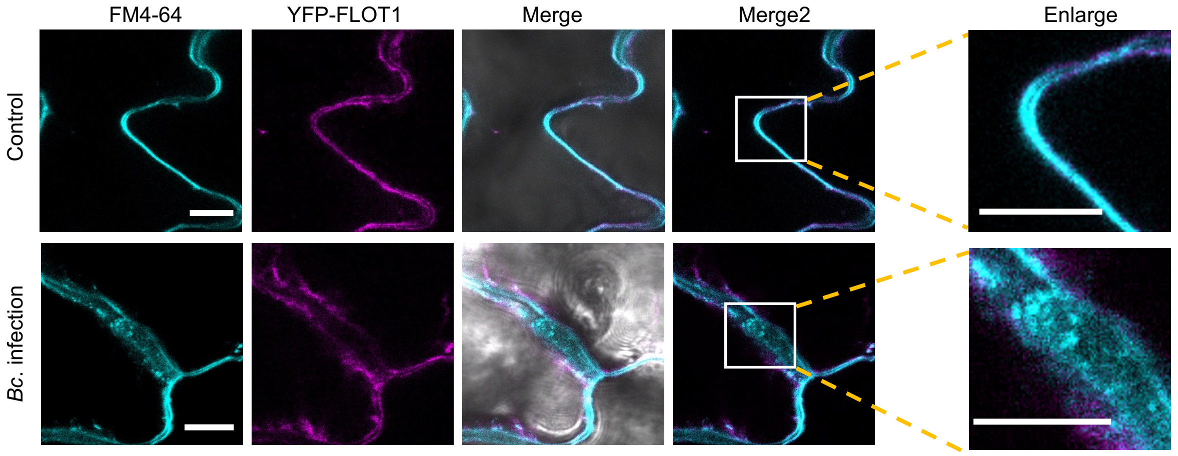

Fig. 6c

Replicate 2

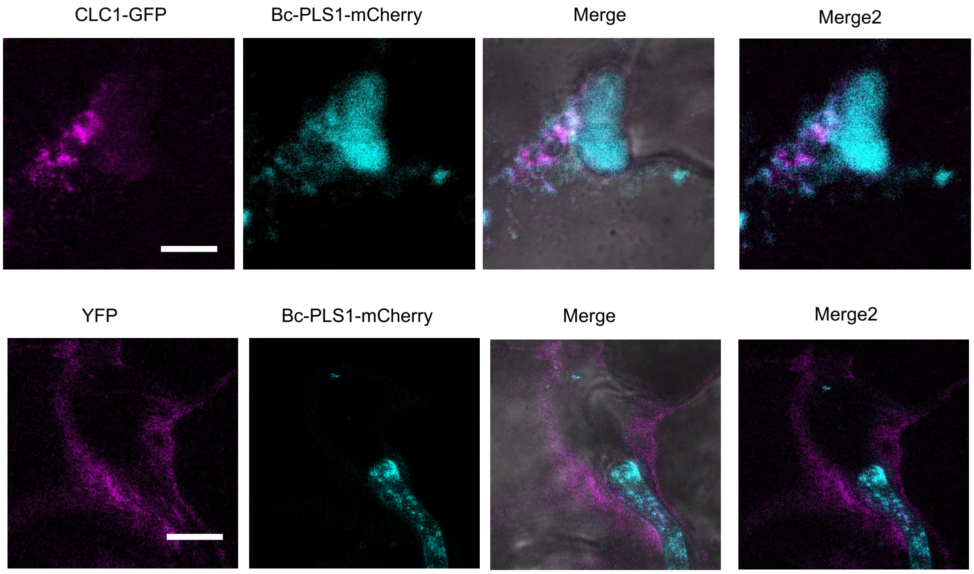

Replicate 3

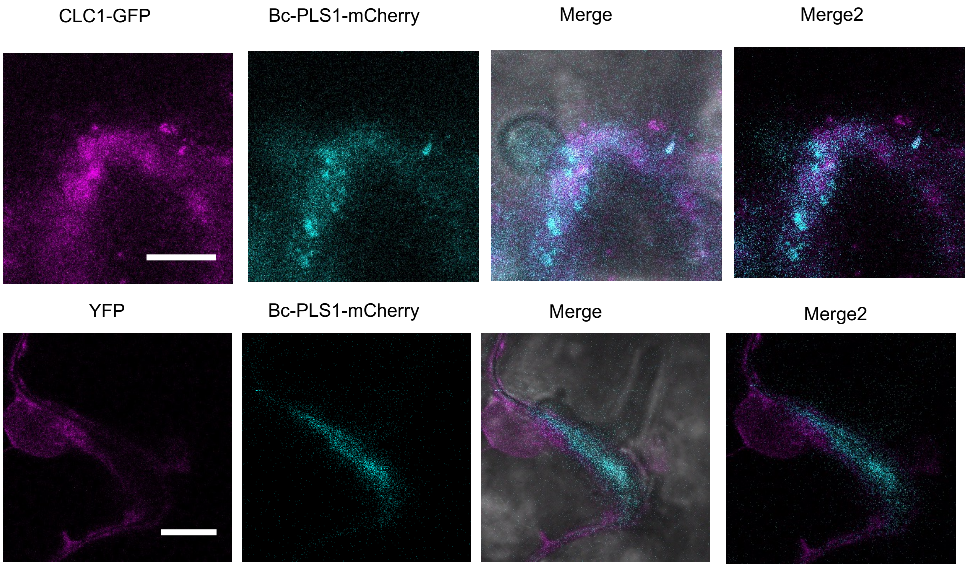

Fig. 6d

Replicate 2

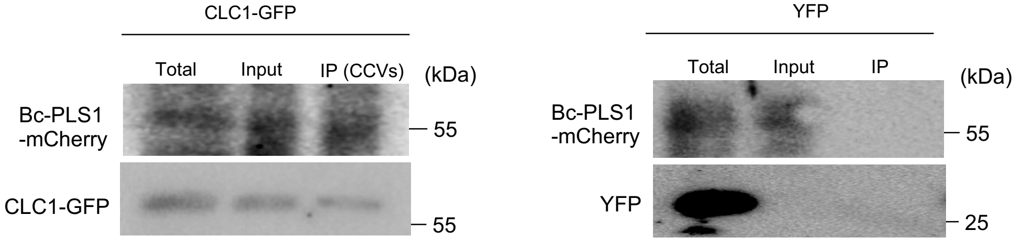

Replicate 3

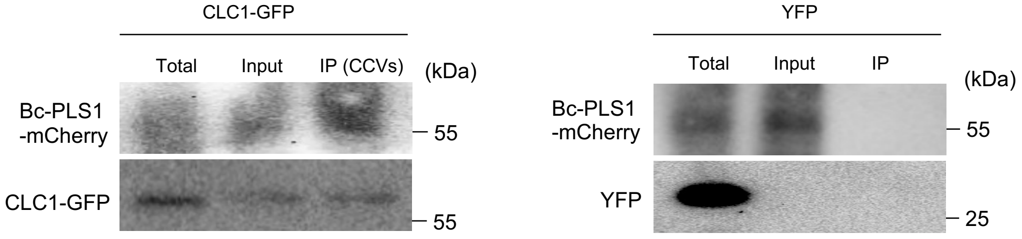

Fig. 6e

Replicate 2

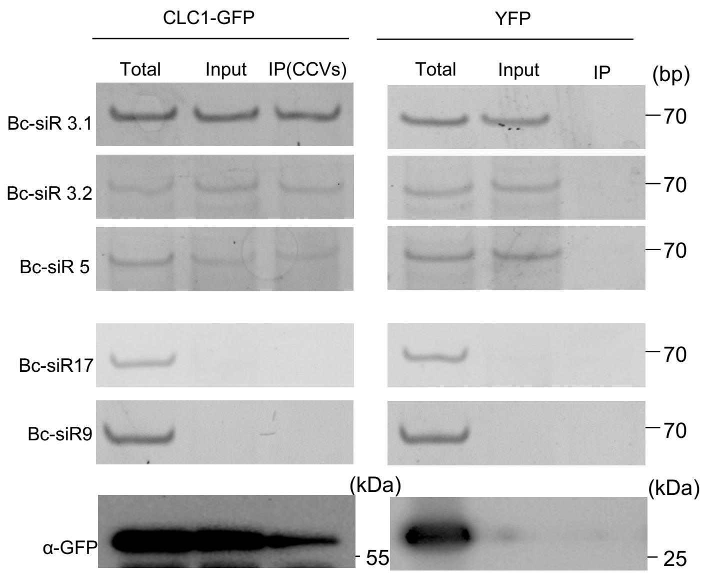

Replicate 3

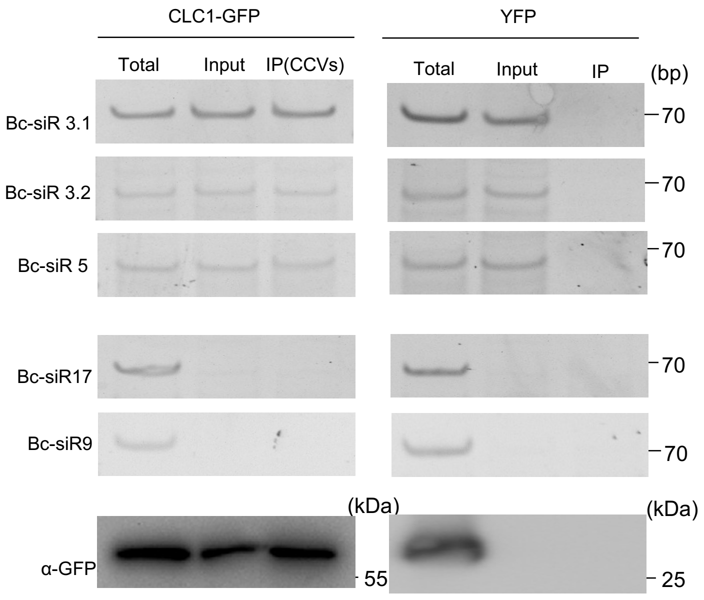

Supplementary Fig. 2c

Replicate 2

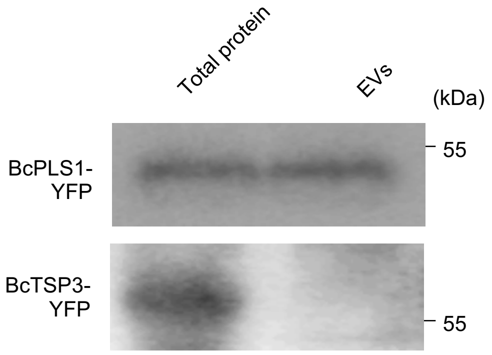

Replicate 3

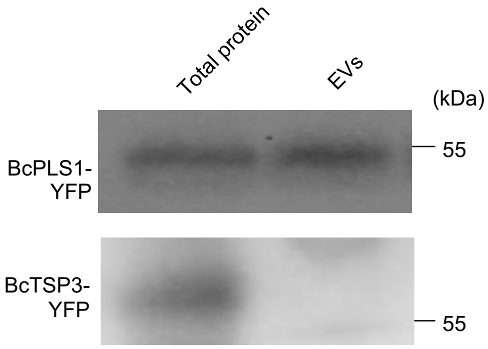

Supplementary Fig. 4a,b

Replicate 2

a

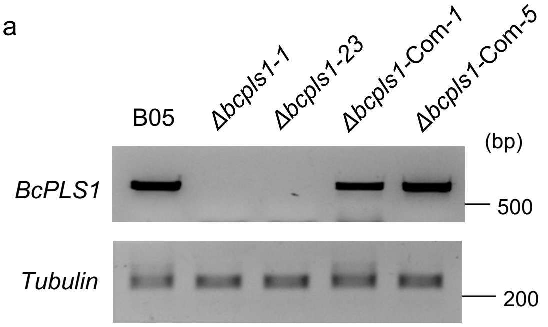

b

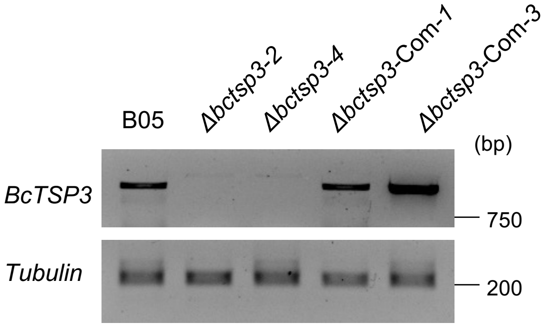

Replicate 3

a

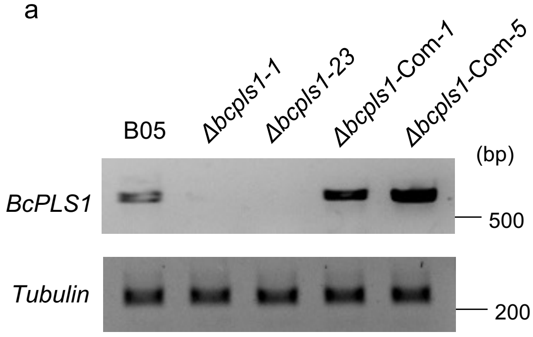

b

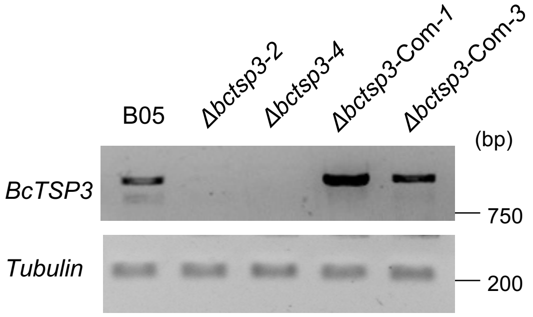

Supplementary Fig. 6a

Replicate2

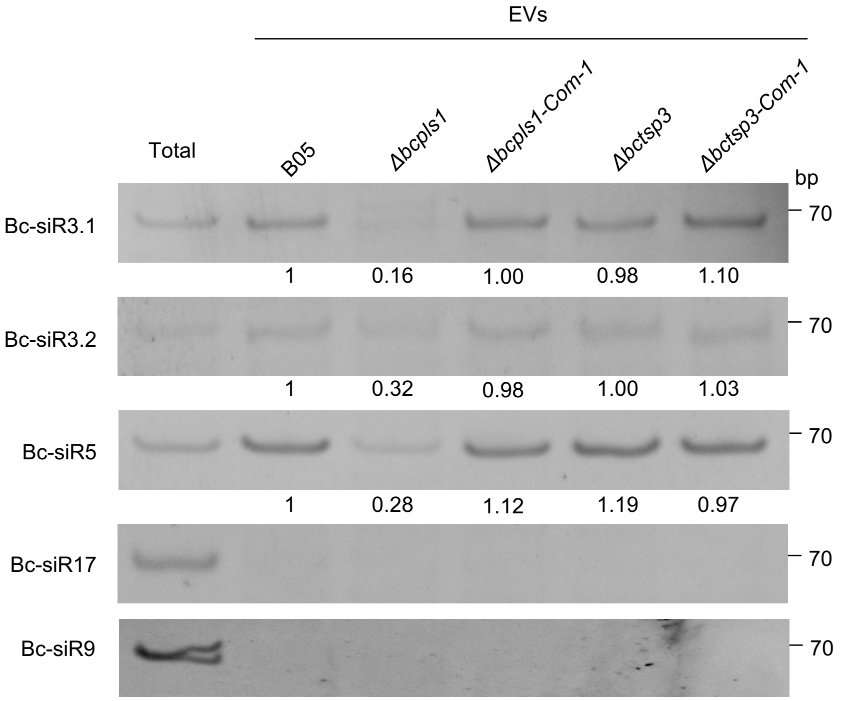

Replicate3

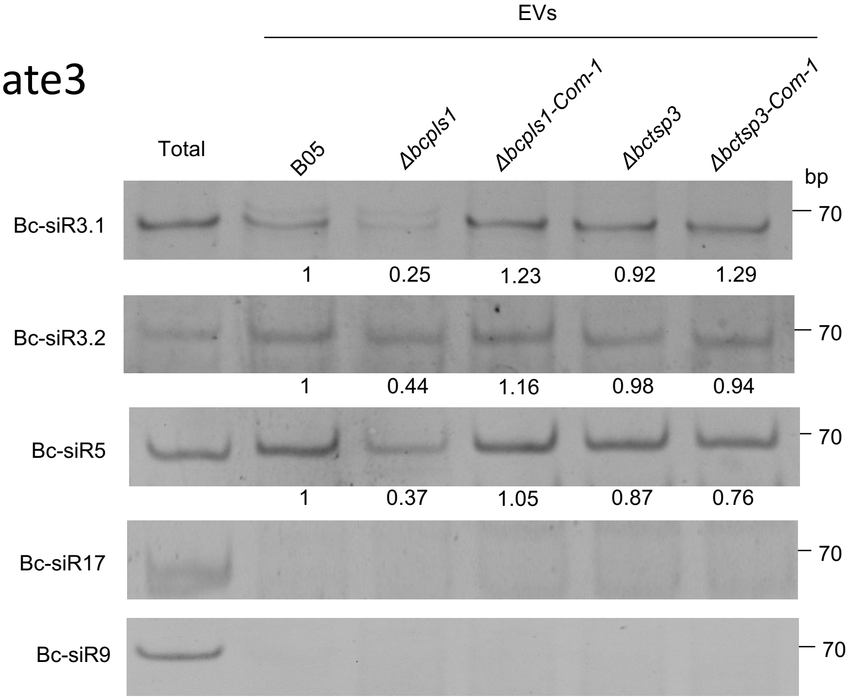

Supplementary Fig. 9c

Replicate2

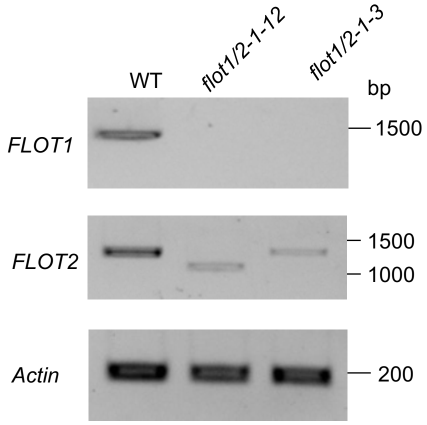

Replicate3

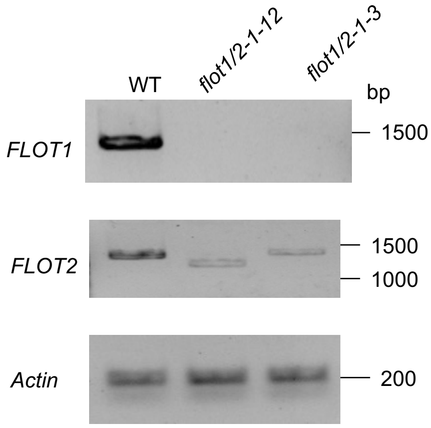

Supplementary Fig. 10

Replicate2

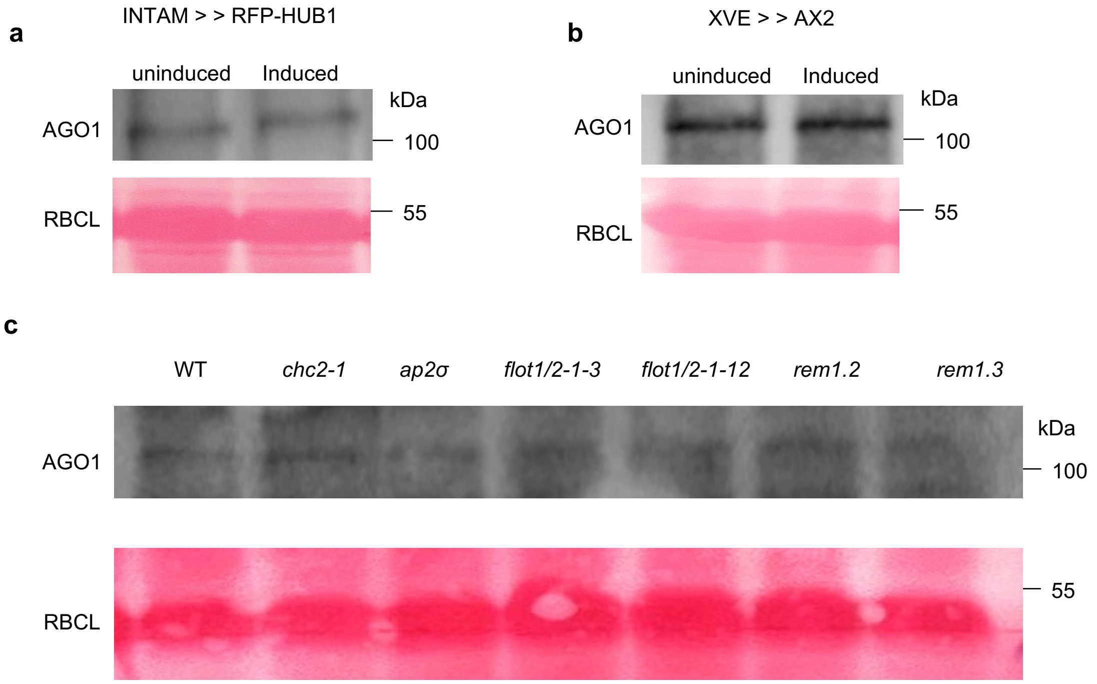

Replicate3

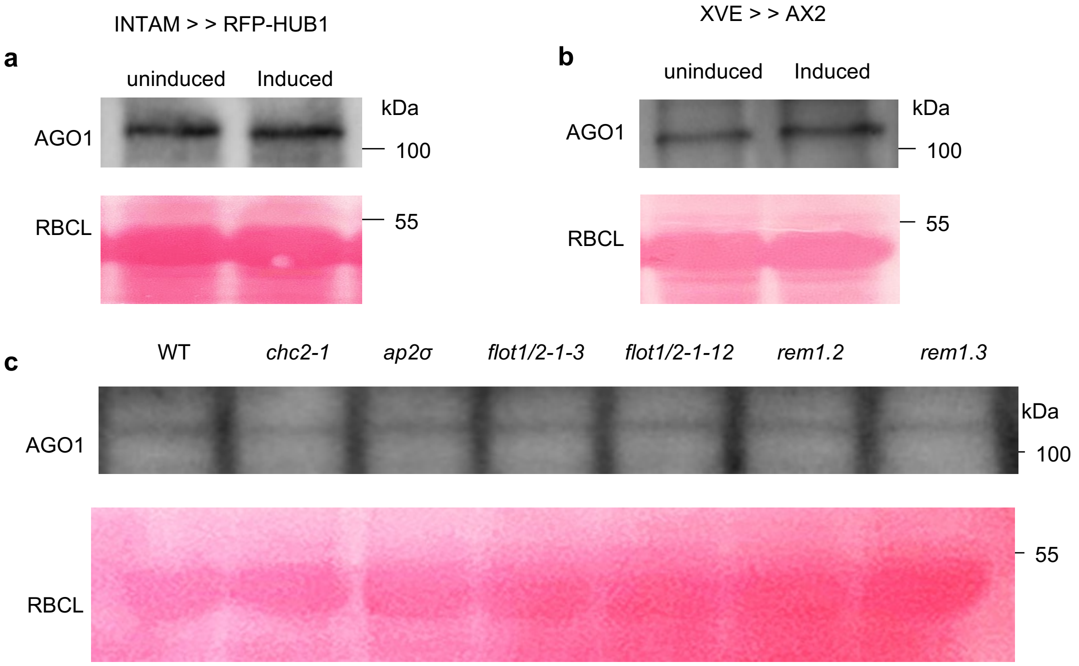

Supplement: Supplementary file 1 — Supplementary Information [file 41467_2023_40093_MOESM1_ESM.pdf]
