# Supplementary material for: Fungal small RNAs ride in extracellular vesicles to enter plant cells through clathrin-mediated endocytosis
Source: Nat Commun. 2023 Jul 20;14:4383. doi: 10.1038/s41467-023-40093-4 (PMC10359353; doi:10.1038/s41467-023-40093-4)
Supplement: Supplementary file 3 — Description of Additional Supplementary Files [file 41467_2023_40093_MOESM3_ESM.pdf]

Description of Additional Supplementary Files

Supplementary Data 1

Description: Primers used in this study.
