# Supplementary material for: Fungal small RNAs ride in extracellular vesicles to enter plant cells through clathrin-mediated endocytosis
Source: Nat Commun. 2023 Jul 20;14:4383. doi: 10.1038/s41467-023-40093-4 (PMC10359353; doi:10.1038/s41467-023-40093-4)
Supplement: Supplementary file 5 — Reporting Summary [file 41467_2023_40093_MOESM5_ESM.pdf]

nature portfolio

Corresponding author(s): Hailing Jin

Last updated by author(s): Jun 27, 2023

Reporting Summary

Nature Portfolio wishes to improve the reproducibility of the work that we publish. This form provides structure for consistency and transparency in reporting. For further information on Nature Portfolio policies, see our Editorial Policies and the Editorial Policy Checklist.

Statistics

For all statistical analyses, confirm that the following items are present in the figure legend, table legend, main text, or Methods section.

n/a	Confirmed	
☐	☒	The exact sample size (*n*) for each experimental group/condition, given as a discrete number and unit of measurement
☐	☒	A statement on whether measurements were taken from distinct samples or whether the same sample was measured repeatedly
☐	☒	The statistical test(s) used AND whether they are one- or two-sided<br>*Only common tests should be described solely by name; describe more complex techniques in the Methods section.*
☒	☐	A description of all covariates tested
☒	☐	A description of any assumptions or corrections, such as tests of normality and adjustment for multiple comparisons
☐	☒	A full description of the statistical parameters including central tendency (e.g. means) or other basic estimates (e.g. regression coefficient) AND variation (e.g. standard deviation) or associated estimates of uncertainty (e.g. confidence intervals)
☐	☒	For null hypothesis testing, the test statistic (e.g. *F*, *t*, *r*) with confidence intervals, effect sizes, degrees of freedom and *P* value noted<br>*Give P values as exact values whenever suitable.*
☒	☐	For Bayesian analysis, information on the choice of priors and Markov chain Monte Carlo settings
☒	☐	For hierarchical and complex designs, identification of the appropriate level for tests and full reporting of outcomes
☒	☐	Estimates of effect sizes (e.g. Cohen's *d*, Pearson's *r*), indicating how they were calculated

Our web collection on statistics for biologists contains articles on many of the points above.

Software and code

Policy information about availability of computer code

Data collection	NanoSight NS300 (Nanoparticle tracking analysis).<br>TEM Talos 120 (transmission electron microscopy).<br>Leica TCS SP5P (Confocal image).<br>Nova NanoSEM 450 (FEI) (Scanning electron microscope)
Data analysis	NanoSight NTA software version 3.1 was used to calculate particle size and numbers.<br>LAS AF was used in this study to analyze the confocal image captured from Leica TCS SP5P confocal microscope.<br>ImageJ was used for measuring lesion size and gray scales.<br>GraphPad Prism version 9.0.2 was used for statistical analyses.

For manuscripts utilizing custom algorithms or software that are central to the research but not yet described in published literature, software must be made available to editors and reviewers. We strongly encourage code deposition in a community repository (e.g. GitHub). See the Nature Portfolio guidelines for submitting code & software for further information.

Data

Policy information about availability of data

All manuscripts must include a data availability statement. This statement should provide the following information, where applicable:
- Accession codes, unique identifiers, or web links for publicly available datasets
- A description of any restrictions on data availability
- For clinical datasets or third party data, please ensure that the statement adheres to our policy

The data that support the findings of this study are available within the paper, Supplementary Information, and Source Data.

Human research participants

Policy information about studies involving human research participants and Sex and Gender in Research.

Reporting on sex and gender	N/A
Population characteristics	N/A
Recruitment	N/A
Ethics oversight	N/A

Note that full information on the approval of the study protocol must also be provided in the manuscript.

Field-specific reporting

Please select the one below that is the best fit for your research. If you are not sure, read the appropriate sections before making your selection.

☒ Life sciences ☐ Behavioural & social sciences ☐ Ecological, evolutionary & environmental sciences

For a reference copy of the document with all sections, see nature.com/documents/nr-reporting-summary-flat.pdf

Life sciences study design

All studies must disclose on these points even when the disclosure is negative.

Sample size	Required experimental sample sizes were estimated based on our previous established protocols (Cai, Q. et al. Plants send small RNAs in extracellular vesicles to fungal pathogen to silence virulence genes. Science, 2018,360, 1126-1129). For EV isolation from fungal culture supernatant, 100ml of YEPD was used to culture B. cinerea spores for 48 hours at room temperature with shaking. The isolated fungal EVs can be used as one set for TEM, nanoparticle tracking analysis or small RNA detection. For CCV isolation, 50 grams of Arabidopsis leaves were used as one set of experiment. The sample sizes were adequate as the experimental results were reproducible.
Data exclusions	No data were excluded from the analysis.
Replication	The number of replicates is indicated in the manuscript. Main conclusions were confirmed in different assays (Immunoblot assay, Immuno-Isolation assay, genetic assay). All the experiments were repeated at least three times independently with similar results.
Randomization	Samples of the same genotypes were randomly collected and pooled for downstream experiments.
Blinding	Experiments were not blinded because no change in results was obtained in different experiments that performed by 2 to 3 coauthors using independent samples. Data were always collected according to the genotypes of the plants.

Reporting for specific materials, systems and methods

We require information from authors about some types of materials, experimental systems and methods used in many studies. Here, indicate whether each material, system or method listed is relevant to your study. If you are not sure if a list item applies to your research, read the appropriate section before selecting a response.

Materials & experimental systems

n/a	Involved in the study	
☐	☒	Antibodies
☒	☐	Eukaryotic cell lines
☒	☐	Palaeontology and archaeology
☒	☐	Animals and other organisms
☒	☐	Clinical data
☒	☐	Dual use research of concern

Methods

n/a	Involved in the study	
☒	☐	ChIP-seq
☒	☐	Flow cytometry
☒	☐	MRI-based neuroimaging

Antibodies

Antibodies used

Mouse monoclonal anti-GFP (Sigma-Aldrich, Cat#11814460001, dilution 1:2,000).
Rabbit polyclonal anti-AGO1 (Science, 2013, 342:6154,118-123, dilution 1:2,000).
Rabbit polyclonal anti-mcherry (abcam, Ca#ab167453, dilution 1:2000).

Validation

Mouse monoclonal anti-GFP was used to detect GFP, YFP and CFP tagged proteins, it is validated for western blot in Supplemental Figure 11 of Chi C Wong et al. "Defective ribosome assembly in Shwachman-Diamond syndrome. Blood. 2011;118(16):4305-4312" and in our manuscript Figure 1b.

Rabbit polyclonal anti-AGO1 was validated for western blot in Figure 3A of Weiberg A et al. "Fungal small RNAs suppress plant immunity by hijacking host RNA interference pathways. Science. 2013;342(6154):118-123"

Rabbit polyclonal anti-mcherry was validated for western blot by manufacture (https://www.abcam.com/mcherry-antibodyab167453.html#lb). mCherry protein was expressed in HEK293 cells, the total cell extraction was separated on SDS-PAGE and blotted with rabbit polyclonal anti-mCherry (abcam, Ca#ab167453) at a dilution of 1:1000. mcherry protein signal can be detected by western blot.
